# Orally administered titanium carbide nanosheets as anti-inflammatory therapy for colitis

**DOI:** 10.7150/thno.70668

**Published:** 2022-05-09

**Authors:** Linqian Hou, Fei Gong, Bo Liu, Xiaoyuan Yang, Linfu Chen, Guangqiang Li, Yuehan Gong, Chao Liang, Nailin Yang, Xian Shen, Zhuang Liu, Liang Cheng

**Affiliations:** 1 Institute of Functional Nano & Soft Materials (FUNSOM), Jiangsu Key Laboratory for Carbon-Based Functional Materials & Devices, Soochow University, Suzhou 215123, China; 2 College of Science, State Key Laboratory of Agricultural Microbiology, Huazhong Agricultural University, Wuhan, 430070, China; 3 Department of Gastrointestinal Surgery, The Second Affiliated Hospital, Wenzhou Medical University, Wenzhou, Zhejiang Province, China

**Keywords:** Ti_3_C_2_ nanosheets, ROS scavenging, oral administration, anti-inflammation, IBD therapy

## Abstract

**Rationale:** Oxidative stress, resulting from excessive reactive oxygen species (ROS), plays an important role in the initiation and progression of inflammatory bowel disease (IBD). Therefore, developing novel strategies to target the disease location and treat inflammation is urgently needed.

**Methods:** Herein, we designed and developed a novel and effective antioxidant orally-administered nanoplatform based on simulated gastric fluid (SGF)-stabilized titanium carbide MXene nanosheets (Ti_3_C_2_ NSs) with excellent biosafety and multiple ROS-scavenging abilities for IBD therapy.

**Results:** This broad-spectrum and efficient ROS scavenging performance was mainly relied on the strong reducibility of Ti-C bound. Intracellular ROS levels confirmed that Ti_3_C_2_ NSs could efficiently eliminate excess ROS against oxidative stress-induced cell damage. Following oral administration, negatively-charged Ti_3_C_2_ NSs specifically adsorbed onto the positively-charged inflamed colon tissue via electrostatic interaction, leading to efficient therapy of dextran sulfate sodium salt (DSS)-induced colitis. The therapeutic mechanism mainly attributed to decreased ROS levels and pro-inflammatory cytokine secretion, and increased M2-phenotype macrophage infiltration and anti-inflammatory cytokine secretion, efficiently inhibiting inflammation and alleviating colitis symptoms. Due to their excellent ROS-scavenging performance, Ti_3_C_2_-based woundplast also promoted skin wound healing and functional vessel formation.

**Conclusions:** Our study introduces redox-mediated antioxidant MXene nanoplatform as a novel type of orally administered nanoagents for treating IBD and other inflammatory diseases of the digestive tract.

## Introduction

Inflammatory bowel disease (IBD), with two main clinical forms, including ulcerative colitis (UC) and Crohn's disease, is a chronic inflammatory disorder of the gastrointestinal tract. Its prevalence adversely affects millions of people around the world 1-3. Though IBD deaths are low, IBD patients are at an increased risk of suffering more serious diseases like colon cancer 4, 5. Currently, small molecular drugs and antibiotics are the treatment options, but these strategies cannot completely cure IBD due to the lack of specificity, and further increasing the drug dose causes severe side-effects 6-8. Consequently, many IBD-targeted therapies rely on pH-, pressure-, and time-dependent therapeutic systems 9. However, most of these therapeutic systems are delivered via the bloodstream, targeting the entire colon, and few are specific to the inflamed colonic lesions 10. Therefore, developing novel strategies to target the disease location and treat inflammation is urgently needed.

During the progression of IBD, toxic reactive oxygen species (ROS) cause biomolecular damage, such as lipid peroxidation, DNA damage, and protein denaturation, thereby triggering high oxidative stress and excessive inflammatory response 11-13. Also, the presence of positively charged proteins in the inflamed colon lesions has been reported 11, 14, 15. Thus, developing novel therapeutic systems with negative charge to target positively charged proteins via electrostatic interactions and eliminating excess ROS for alleviating inflammation was an extremely effective treatment for IBD. For instance, multi-enzymes encapsulated into liposomes, montmorillonite-anchored ceria nanoparticles, and redox nanoparticles with negative charge were successfully constructed to scavenge ROS for IBD therapy 16-19. However, the instability of natural enzymes, nanozymes, or other therapeutic agents in the harsh, acidic, and enzyme-rich environment encountered in the complex gastrointestinal tract may reduce their pharmacological activities and affect *in vivo* performance 20. Consequently, innovative antioxidant strategies are urgently required to effectively prevent and resolve intestinal inflammation in IBD.

**Scheme 1:**
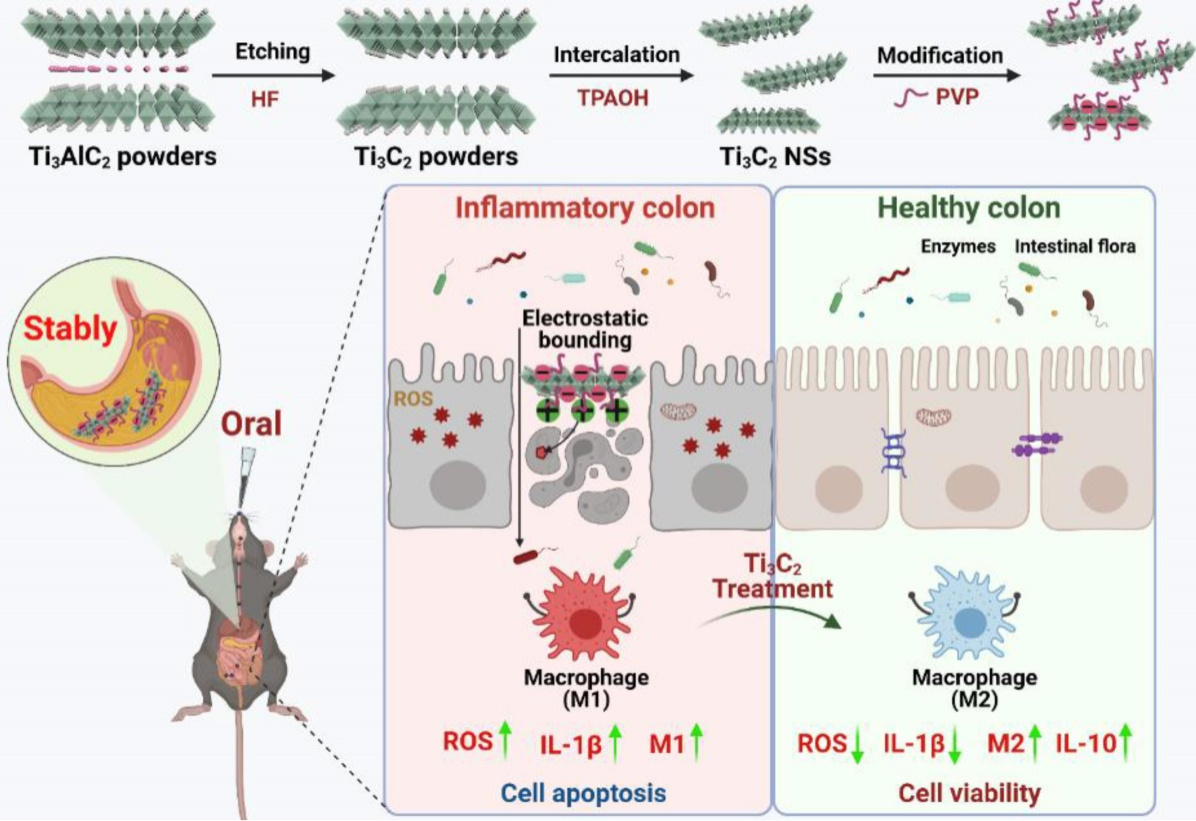
** Schematic illustration shows the preparation and application of Ti_3_C_2_ NSs.** The route of Ti_3_C_2_ MXene prepared from the Ti_3_AlC_2_ MAX phase and the surface modification of them by PVP polymer. After oral administration of gastric acid-tolerant Ti_3_C_2_ NSs, they could bind to the inflamed mucosa through electrostatic force in the colon. Ti_3_C_2_ NSs could scavenge the excess reactive oxygens species (ROS) and regulate the phenotype of macrophages at the inflammation site, and thus relieve inflammatory bowel disease (IBD).

Currently, two-dimensional titanium carbide MXene nanosheets (2D Ti_3_C_2_ NSs) with ultrathin layer-structured topology have attracted much attention for biomedical applications 21, 22. In this context, Ti_3_C_2_ NSs with intrinsic Ti-C redox-active sites show profound chemical reactivity toward ROS, and can serve as an efficient antioxidant to treat many ROS-related diseases, including IBD 23. Due to their specificity, stability, and good ROS scavenging, we employed Ti_3_C_2_ NSs as novel orally administered ROS scavengers specifically targeted to the inflamed colon lesions via electrostatic interactions for IBD treatment (Scheme 1). Ti_3_C_2_ NSs were synthesized by a two-step exfoliation method and exhibited broad-spectrum redox-mediated ROS scavenging activity. The intracellular ROS levels confirmed that Ti_3_C_2_ NSs could efficiently eliminate excess ROS against oxidative stress-induced cell damage. Moreover, the negatively-charged Ti_3_C_2_ NSs could be specifically adsorbed onto positively-charged inflamed colon tissue after oral administration, leading to efficient therapy of dextran sulfate sodium salt (DSS)-induced colitis. The therapeutic mechanism was mainly attributed to decreased ROS levels and pro-inflammatory cytokine secretion and increased M2-phenotype macrophage infiltration and anti-inflammatory cytokine secretion, efficiently inhibiting inflammation and alleviating colitis symptoms. Additionally, Ti_3_C_2_-based woundplasts have been employed to promote skin wound healing and functional vessel formation due to their excellent ROS scavenging ability. Thus, our study highlights an effective strategy based on 2D Ti_3_C_2_ NSs to treat IBD disease via redox-mediated antioxidative protection.

## Results and Discussion

**Figure 1:**
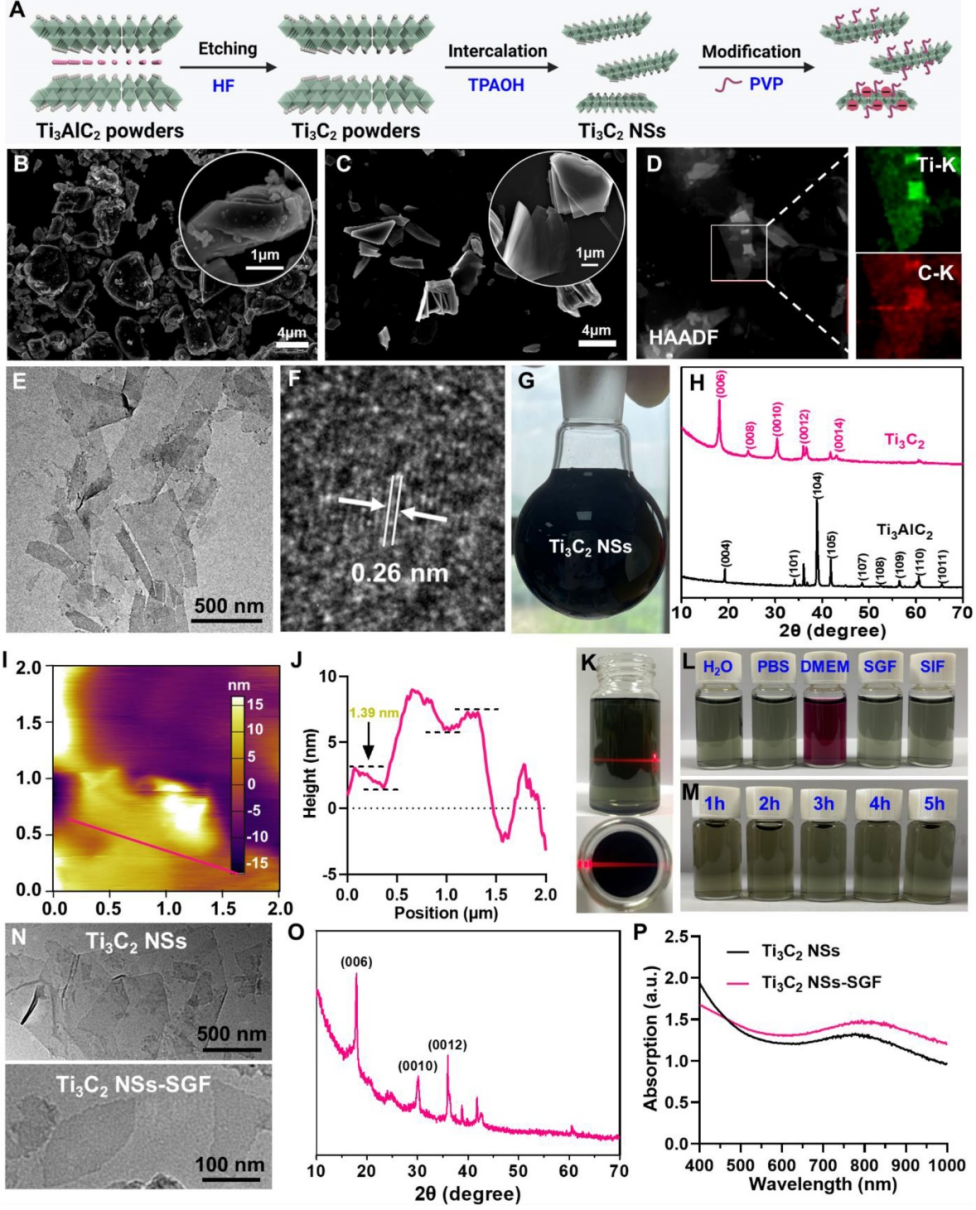
** Synthesis and characterization of 2D ultrathin Ti_3_C_2_ NSs (MXenes).** (**A**) Scheme shows the synthesis procedures of Ti_3_C_2_ NSs and their surface modification. (**B-C**) SEM images of Ti_3_AlC_2_ powders and TPAOH-intercalated Ti_3_C_2_ (inset is the corresponding high-magnification SEM images). (**D**) High-angle annular dark-field scanning TEM (HAADF-STEM) image of Ti_3_C_2_ NSs and the element distribution of Ti (green) and C (red) onside the Ti_3_C_2_ NSs. (**E-F**) TEM and high-resolution TEM (HRTEM) images of as-synthesized Ti_3_C_2_ NSs. (**G**) A photograph of Ti_3_C_2_ NSs in water (2 mg/mL). (**H**) XRD spectra of the Ti_3_AlC_2_ MAX phase and Ti_3_C_2_ Mxene. (**I-J**) AFM image of the Ti_3_C_2_ NSs and the height profiles along the red lines. (**K**) The obvious Tyndall effect of the Ti_3_C_2_ NSs in H_2_O. (**L**) The stability of the PVP-modified Ti_3_C_2_ NSs in various buffers including H_2_O, PBS, DMEM, SGF, and SIF. (**M**) The stability of the PVP-modified Ti_3_C_2_ NSs in SGF for 1 h, 2 h, 3 h, 4 h, and 5 h. (**N-P**) TEM images, XRD spectrum, and UV-vis-NIR spectra of Ti_3_C_2_ NSs before and after incubation in SGF for 5 h.

2D Ti_3_C_2_ NSs were synthesized via a two-step exfoliation strategy based on hydrofluoric acid (HF) etching and tetrapropylammonium hydroxide (TPAOH) intercalation of titanium aluminum carbide (Ti_3_AlC_2_) powder (Figure 1A) 24-26. Initially, the Ti_3_AlC_2_ MAX phase with a layered morphology was etched with 20% HF solution for 4 h to remove Al layers. Subsequently, TPAOH aqueous solution was employed to insert the etched Ti_3_C_2_ flakes to reduce the sheet thickness (Figure 1B-C). The transmission electron microscopy (TEM) image revealed that the synthesized Ti_3_C_2_ NSs exhibited typical sheet-like morphology with an average size of ~500 nm (Figure 1E). The lattice spacing of ~0.26 nm obtained from the high-resolution TEM (HRTEM) image corresponded with the standard Ti_3_C_2_ (JCPDS No. 65-0242) (Figure 1F-H). The energy dispersive spectrometer (EDS) elemental mapping displayed the homogeneous distribution of Ti and C elements in Ti_3_C_2_ NSs (Figure 1D). As a sheet morphology, the height of Ti_3_C_2_ NSs determined by atomic force microscopy (AFM) was ~1.39 nm (Figure 1I-J). Importantly, this two-step exfoliation strategy could be scaled-up to prepare large amounts of high quality Ti_3_C_2_ NSs that could be well dispersed in water for a long-term storage (Figure 1G,1K-L). However, they would aggregate in the salt solution and showed poor biocompatibility (Figure S1). Therefore, polyvinylpyrrolidone (PVP, MW 10k) polymer was used to stabilize Ti_3_C_2_ NSs via the steric hindrance among the macromolecular chains. Typical C=O and C-N signals in the Ti_3_C_2_-PVP sample were observed by Fourier transform infrared (FTIR) spectroscopy, indicating successful PVP modification onto the surface of Ti_3_C_2_ NSs (Figure S2). The amount of PVP modified on the surface of Ti_3_C_2_ NSs measured by thermogravimetric analysis (TGA) was ~12.4% (Figure S3). The PVP-modified Ti_3_C_2_ NSs exhibited excellent dispersity and long-term colloidal stability without significant dimensional change in H_2_O, phosphate-buffered saline (PBS), and Dulbecco's Modified Eagle Medium (DMEM) (Figure 1L, Figure S4). Importantly, these Ti_3_C_2_-PVP NSs showed excellent long-time stability in simulated gastric fluid (SGF, pH = 1.5) and simulated intestinal fluid (SIF, pH = 6.8), indicating their suitability for oral administration. TEM and X-ray diffraction (XRD) characterization showed no change in the morphology and nanostructure of Ti_3_C_2_-PVP NSs after 5 h incubation in SGF (Figure 1M-O). Also, the UV-vis-NIR absorption spectra of Ti_3_C_2_-PVP NSs and SGF-incubated Ti_3_C_2_-PVP NSs showed no noticeable difference, further verifying the excellent acid corrosion resistance of Ti_3_C_2_-PVP NSs (Figure 1P). Finally, we investigated the surface potential of Ti_3_C_2_ NSs after incubation with SGF, and found that SGF-treated Ti_3_C_2_ NSs still had a negative charge (Figure S5), confirming the strong stability of Ti_3_C_2_ NSs in SGF. Together, these results verified the acid stability of Ti_3_C_2_ NSs, which could be used as a novel oral administration for treating inflammatory diseases of the digestive tract.

**Figure 2:**
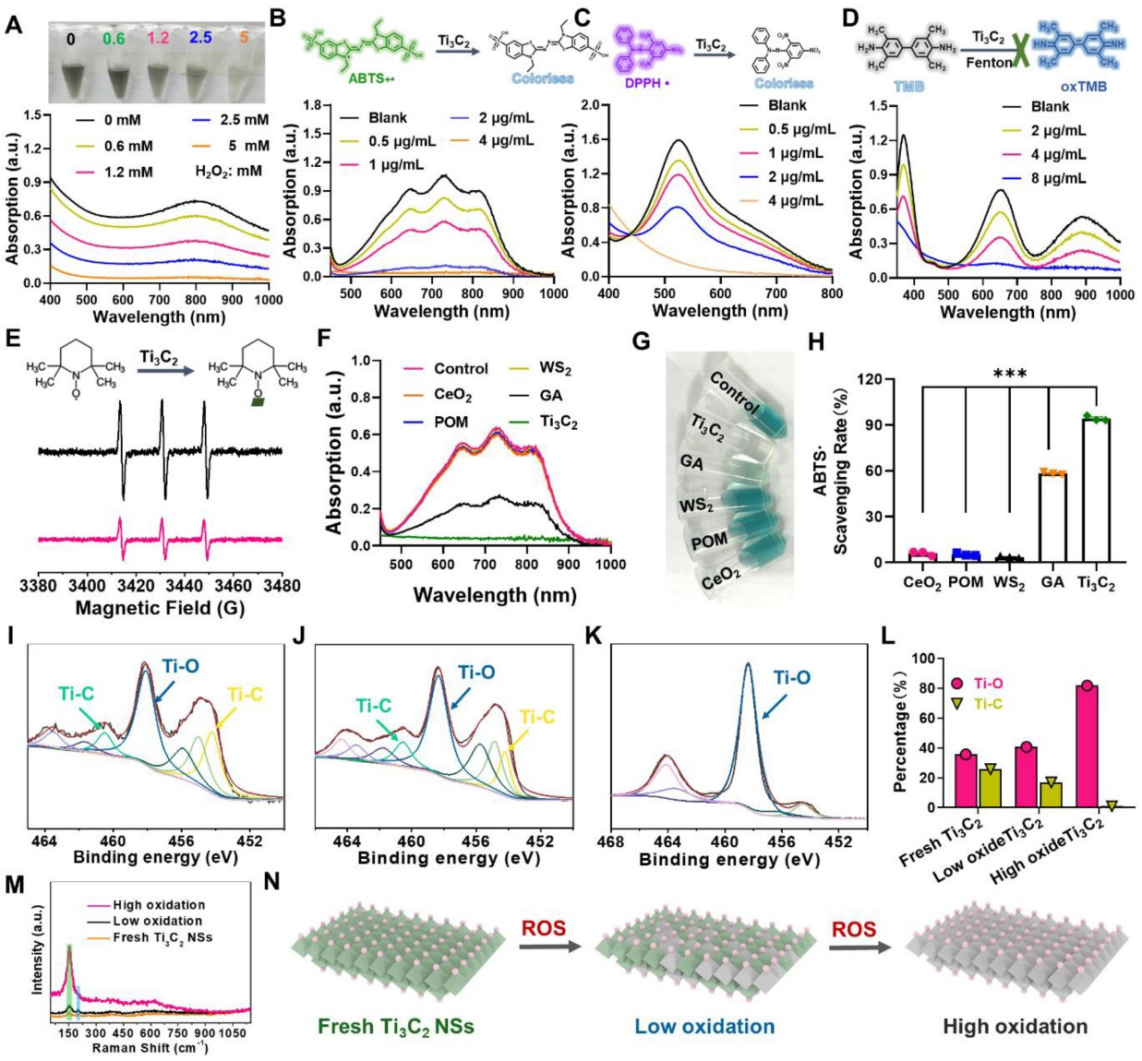
** The performance and the probable mechanism of ROS scavenging by Ti_3_C_2_ NSs.** (**A**) UV-vis absorbance spectra of H_2_O_2_-treated Ti_3_C_2_ NSs (H_2_O_2_ concentrations: 0.6, 1.2, 2.5, and 5 mM). (**B-C**) UV-vis absorbance spectra of ABTS+• and DPPH• radicals after incubation with Ti_3_C_2_ NSs at different concentrations (0.5, 1, 2, and 4 μg/mL). (**D**) UV-vis absorbance spectra of TMB after co-incubation with Fenton agent and Ti_3_C_2_ NSs (Fe^2+^: 10 μM, H_2_O_2_: 50 μM, TMB: 0.3 mM; Ti_3_C_2_: 2, 4, and 8 μg/mL). (**E**) ESR spectra of TEMPO indicating radical scavenging ability of Ti_3_C_2_ NSs. (**F**) UV-vis absorbance spectra of ABTS+• radical after incubation with same concentrations of the CeO_2_, POM, WS_2_, GA and Ti_3_C_2_ NSs for 1 min (dose: 2 μg/mL). **(G)** A photograph of the above-mentioned solutions after reaction. **(H)** The quantitative analysis of ABTS+• scavenging rate by them. (**I-L**) XPS spectra of Ti 2P from Ti_3_C_2_ NSs with different degree of oxidation and corresponding peak area. (**M**) Raman spectra of Ti_3_C_2_ NSs with different degree of oxidation. (**N**) Schematic diagram of the antioxidant mechanism of Ti_3_C_2_ NSs. The statistical significance was calculated by ordinary one-way ANOVA. *P < 0.05; **P < 0.01; ***P < 0.005; ****P < 0.001.

Considering the key role of reactive oxide/nitrogen species (ROS/RNS) in inflammatory diseases, the potential of Ti_3_C_2_ NSs as ROS/RNS scavengers was evaluated. Initially, the color of Ti_3_C_2_ NSs suspension gradually faded to colorless after incubation with different concentrations of hydrogen peroxide (H_2_O_2_: 0.6, 1.2, 2.5, and 5 mM), and their UV-vis-NIR absorption spectra disappeared as the H_2_O_2_ concentration increased, indicating the reactivity and scavenging ability of Ti_3_C_2_ NSs toward H_2_O_2_ (Figure 2A). The UV-vis-NIR absorption spectra of two intrinsic RNS, 1,1-diphenyl-2-picrylhydrazyl radicals (DPPH•) and 2, 2-azino-bis (3-ethylbenzothiazoline-6-sulfonic acid) radical (ABTS+•), decreased significantly after mixing with Ti_3_C_2_ NSs (0.5, 1, 2, and 4 μg/mL). Also, Ti_3_C_2_ NSs could scavenge almost all of RNS at a low concentration (2 μg/mL), suggesting an excellent radical-scavenging ability of the synthesized Ti_3_C_2_ NSs (Figure 2B-C).

The superior antioxidant activity and stability of Ti_3_C_2_ NSs were confirmed by ABTS+• scavenging experiments (Figure S6-S9). Besides, the ROS-scavenging efficiency of Ti_3_C_2_ NSs was evaluated by hydroxyl radicals (HO•), regarded as one of the strong free radicals frequently involved in cell injuries and body diseases. A peroxidase substrate, 3,3,5,5-tetramethylbenzidine (TMB), used as an HO• indicator (colorless) could be oxidized into *ox*TMB (blue) by HO• (Figure 2D). The blue color of the reaction system sharply faded by adding Ti_3_C_2_ NSs, indicating a significant decrease in the HO•-induced *ox*TMB concentration. Moreover, TMB was not easily oxidized by HO• in the presence of Ti_3_C_2_ NSs at a much lower concentration (8 μg/mL), further indicating an excellent ROS scavenging ability of Ti_3_C_2_ NSs. Also, the terephthalic acid (TA) fluorescence probe, as a specific HO• indicator, confirmed the exceptional HO•-scavenging performance of Ti_3_C_2_ NSs (Figure S10). Lastly, 2,2,6,6-tetramethyl-1-piperidinyloxy (TEMPO, intrinsic free radical) was employed to examine the antioxidative properties of Ti_3_C_2_ NSs by electron spin resonance (ESR) spectra. The results showed that the characteristic peaks of TEMPO could be effectively eliminated by Ti_3_C_2_ NSs, confirming the excellent antioxidative properties of Ti_3_C_2_ NSs (Figure 2E, Figure S11). Taken together, Ti_3_C_2_ NSs exhibited broad-spectrum and efficient ROS-scavenging ability to treat inflammatory diseases.

Then, a series of experiments were performed to understand the excellent anti-oxidant properties of Ti_3_C_2_ NSs and analyze structural changes of Ti_3_C_2_ NSs after ROS scavenging. As revealed by TEM imaging, Ti_3_C_2_ NSs degraded into ultra-small nanodots after reaction with ROS (Figure S12), and XRD spectra showed the disappearance of Ti_3_C_2_ NSs characteristic peaks during this process (Figure S13). X-ray photoelectron spectrometer (XPS) spectra of Ti 2p showed oxidation of Ti_3_C_2_ NSs into Ti-based oxides after elimination of ROS compared with the original Ti_3_C_2_ NSs (Figure 2I-K). The two characteristic Ti-C peaks (460.5 eV and 454.2 eV) almost disappeared, and the peak intensity decreased from 25.7% to 0.9% while the intensity of Ti-O peaks (458.1 eV) significantly increased from 35.7% to 81.7% (Figure 2L), indicating intensive oxidation of Ti_3_C_2_ NSs during ROS scavenging.

We also performed Raman spectrum to analyze the surface change of Ti_3_C_2_ NSs during ROS scavenging. Notably, the characteristic peak of Ti_3_C_2_ NSs at 260 cm^-1^ (namely Ti-C) significantly decreased after ROS treatment, while an enhanced peak at 155 cm^-1^ (Ti-O) was observed in the ROS-treated samples, demonstrating that Ti-O was generated from Ti-C and then accumulated on the surface of Ti_3_C_2_ NSs (Figure 2M, Figure S14-S15) 27-29. The strong ROS scavenging could be explained by the following mechanism: Ti_3_C_2_ NSs scavenge ROS through the oxidation-reduction reactions between Ti-C and ROS, leading to a significant decrease of Ti-C and increase of Ti-O, resulting in efficient ROS scavenging (Figure 2N).

We conducted the ABTS+• radicals assay (Figure 2F-H, Figure S16) to compare the excellent ROS-scavenging performance of Ti_3_C_2_ NSs with the currently reported ROS-scavenging agents, including cerium dioxide (CeO_2_) 17, 30, 31, molybdenum-based polyoxometalate (POM) 32, transition-metal dichalcogenide (WS_2_) 33, and gallic acid (GA) 34. After incubation with the same concentration, the characteristic absorption peak intensity of ABTS+• radicals showed no change in CeO_2_, POM, and WS_2_ groups, a significant decrease in the GA group, and completely disappeared in the Ti_3_C_2_ NSs group (Figure 2F). Also, the color of various treatments revealed similar results (Figure 2G). Quantitative analysis of the ABTS+• scavenging rate further confirmed the comparably higher antioxidant performance of Ti_3_C_2_ NSs than CeO_2_ (~16-fold), POM (~18-fold), WS_2_ (~27-fold), and GA (~1.6-fold) antioxidative agents (Figure 2H).

**Figure 3:**
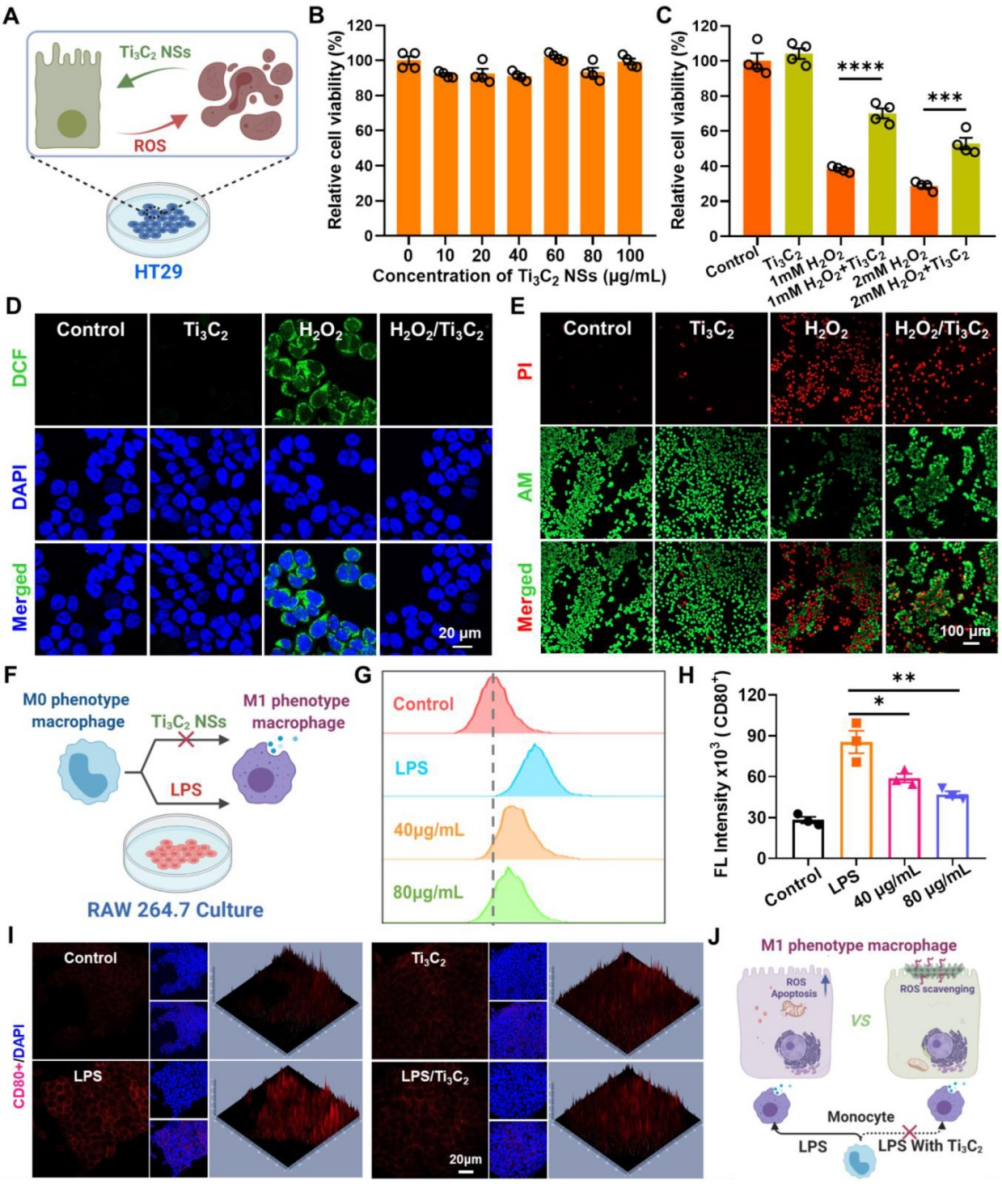
***In vitro* antioxidant investigation by Ti_3_C_2_ NSs.** (**A**) Schematic illustration shows the intracellular ROS scavenging and cell protection by Ti_3_C_2_ NSs. (**B**) Relative viabilities of HT29 cells after incubation with different concentration of Ti_3_C_2_ NSs (0, 10, 20, 40, 60, 80, and 100 μg/mL). (**C**) Relative viabilities of HT29 cells with different treatments including Ti_3_C_2_ (40 μg/mL), H_2_O_2_ (1 or 2 mM), and Ti_3_C_2_ plus H_2_O_2_. (**D**) Confocal images of HT29 cells stained with DCFH-DA after various treatments as indicated. (**E**) Confocal images of HT29 cells stained with Calcein-AM (green, live cells) and propidium iodide (red, dead cells) after treatments. (**F**) Scheme shows that Ti_3_C_2_ NSs could regulate the polarization of macrophages. (**G-H**) Flow cytometric examination and statistical analysis of the M1-phenotype macrophages in different groups. (**I**) Immunofluorescence images indicate the M1-type macrophages in different groups. (**J**) Scheme shows the intracellular ROS scavenging and the Ti_3_C_2_ and the macrophages polarization regulating by Ti_3_C_2_ NSs. The statistical significance was calculated by two-tailed student's t test. *P < 0.05; **P < 0.01; ***P < 0.005; ****P < 0.001.

Next, we studied the intracellular ROS-scavenging performance of Ti_3_C_2_ NSs on human colonic epithelial (HT29) cells, Raw 264.7 macrophage, and human umbilical vein endothelial cells (HUVECs) (Figure 3A). Ti_3_C_2_ NSs exhibited no apparent cytotoxicity to HT29, Raw 264.7 macrophage, and HUVECs cells by the standard methyl thiazolyl tetrazolium (MTT) assay even at a high concentration (100 μg/mL) for 12 h (Figure 3B, Figure S17-S18). HT29 internalized Ti_3_C_2_ NSs via endocytosis in a time-dependent fashion (Figure S19-20). Subsequently, the protection mechanism of Ti_3_C_2_ NSs for H_2_O_2_-induced HT29 apoptosis was evaluated. As revealed by the MTT assay, H_2_O_2_ treatment could amplify the intracellular oxidative stress and eventually cause HT29 apoptosis (Figure 3C). In contrast, pretreatment of Ti_3_C_2_ NSs could significantly relieve oxidative stress and abolish cell apoptosis, indicating that almost all of H_2_O_2_ was scavenged by Ti_3_C_2_ NSs. Furthermore, calcein AM/propidium iodide (AM/PI) staining of H_2_O_2_-treated HT29 showed a red signal indicative of dead cells. However, the red signal significantly decreased upon treatment with Ti_3_C_2_ NSs, indicating that Ti_3_C_2_ NSs could scavenge H_2_O_2_ and block H_2_O_2_-induced cell apoptosis (Figure 3E, Figure S21).

Since oxidative stress induced by excessive ROS generation induces lipid peroxidation, protein damage, and DNA breaks, the intracellular ROS-scavenging performance of Ti_3_C_2_ NSs was further studied via 2,7-dichlorofluorescein diacetate (DCFH-DA) probe, which reacts with intracellular ROS and forms green fluorescent DCF. We observed a strong green fluorescence signal in the H_2_O_2_-treated HT29 cells compared with untreated or only Ti_3_C_2_ NSs-treated cells (Figure 3D, Figure S22). After pretreatment with Ti_3_C_2_ NSs, the DCF fluorescence signal remarkably decreased in H_2_O_2_-treated HT29 cells, suggesting that most of the intracellular H_2_O_2_ was eliminated by Ti_3_C_2_ NSs. Thus, Ti_3_C_2_ NSs showed high *in vitro* ROS-scavenging ability and protective effect against the high oxidative-stress-induced cell death.

Besides the intracellular ROS-scavenging evaluation, the regulatory function of Ti_3_C_2_ NSs involved in macrophage polarization was investigated using lipopolysaccharide (LPS)-treated mouse macrophage cells (RAW 264.7) (Figure 3F). In addition to ROS scavenging, the macrophage phenotype plays a critical role in the pathogenesis of many inflammatory diseases. Macrophages in the inflammatory site are primarily of the M1 phenotype and promote inflammation by secreting various inflammatory cytokines. Therefore, down-regulating the M1 phenotype macrophages is a potential method for treating inflammatory diseases 35-37. To examine the capability of Ti_3_C_2_ NSs for reprogramming macrophages, LPS-pretreated RAW 264.7 cells with the M1 phenotype were analyzed by immunofluorescence staining with the antibody against CD80, an M1 marker. The strong CD80^+^ signal of LPS-pretreated RAW 264.7 cells significantly decreased after treatment with Ti_3_C_2_ NSs (Figure 3I), demonstrating the effective inhibition of macrophage polarization. Furthermore, the flow cytometric analysis combined with the enzyme-linked immunosorbent assay (ELISA) confirmed that Ti_3_C_2_ NS treatment could significantly inhibit the expression of pro-inflammatory factor interleukin-6 (IL-6) and reduce the proportion of LPS-induced inflammatory macrophages (Figure 3G-H, Figure S23). Thus, Ti_3_C_2_ NSs with strong antioxidant properties could efficiently scavenge intracellular ROS and regulate macrophage polarization to treat inflammatory diseases (Figure 3J).

**Figure 4:**
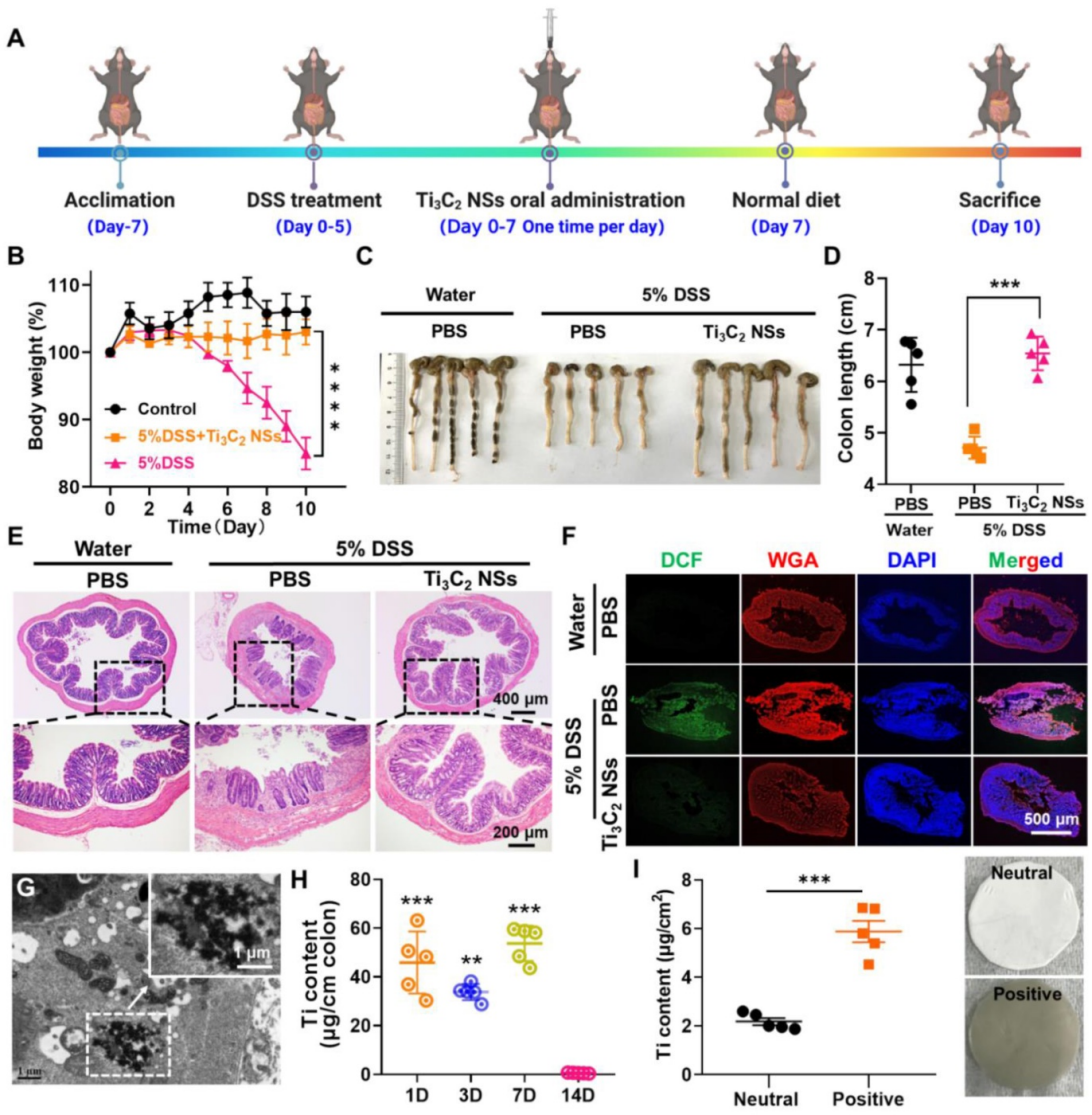
***In vivo* therapeutic effect of Ti_3_C_2_ NSs on DSS-induced colitis.** (**A**) The scheme shows the protocol of DSS-induced colitis and the oral administration of Ti_3_C_2_ NSs for IBD treatment. (**B**) Daily body weight changes of mice from different groups as indicated. (**C-D**) Mice were sacrificed on day 10, and colons were collected, imaged, and their lengths measured. (**E**) Microscopy images of H&E-stained of longitudinal sections of these colon after different treatments. (**F**) Confocal images of colon tissue stained by DCFH-DA from various groups. (**G**) Bio-TEM of colon tissue from these mice after Ti_3_C_2_ NSs oral administration (Green, DCF; WGA, Wheat Germ Agglutini, Red; DAPI, blue). (**H**) The Ti levels in the colon tissue determined by ICP-OES at different days. (**I**) The Ti contents in the neutral and positively charged film after incubation with Ti_3_C_2_ solutions (The insert is the corresponding photograph). The statistical significance was calculated by two-tailed student's t test. *P < 0.05; **P < 0.01; ***P < 0.005; ****P < 0.001.

Many positively charged proteins and excessive ROS were frequently observed in the inflamed colon lesions.^14^ Negatively charged nanoplatforms targeting positively charged proteins and scavenging excess ROS represent an extremely effective treatment for alleviating inflammation. We used negatively charged Ti_3_C_2_ NSs to target positively charged colonic lesions. A positively charged film of methanol-activated PVDF (polyvinylidene fluoride) membranes was employed to simulate the inflamed colon tissue and evaluate the charge-attracted targeting accumulation of Ti_3_C_2_ NSs. After incubation with Ti_3_C_2_ NSs, the positively charged film showed black color, indicating that Ti_3_C_2_ NSs aggregated on the surface through charge adsorption (Figure 4I). Importantly, Ti_3_C_2_ NS concentration on the positively-charged film was 3-fold compared with the neutral film, confirming the effective electrostatic targeting of the negatively-charged Ti_3_C_2_ NSs to the inflamed colon.

The efficient ROS-scavenging property, excellent stability in SGF, and the inflamed colon targeting capability prompted us to investigate the *in vivo* therapeutic efficacy of Ti_3_C_2_ NSs for the treatment of inflammatory bowel disease (IBD) via oral administration. Mice were given 5% dextran sulfate sodium salt (DSS) supplemented in the drinking water for 5 consecutive days to establish an IBD model, and Ti_3_C_2_ NSs (45 mg/kg) were orally administered for one week according to the previous dose climbing experiments (Figure 4A, Figure S24). The body weight changes were monitored daily to evaluate the therapeutic efficacy (Figure 4B). Compared with their initial weight, healthy mice showed an increased body weight; however, DSS-induced colitis mice showed ~15.4% decreased body weight on day 10, indicating the successful establishment of the IBD model. After the oral administration of Ti_3_C_2_ NSs, the body weight of the colitis mice slightly increased, demonstrating the positive therapeutic outcome of Ti_3_C_2_ NSs for IBD. Also, compared with the clinical drug 5-aminosalicylic acid (5-ASA), Ti_3_C_2_ NSs exhibited a satisfactory therapeutic effect after oral administration (Figure S25). We also examined pathological colon sections and measured changes in the colon length to evaluate the therapeutic efficacy of the Ti_3_C_2_ NSs. DSS-induced colitis shortened the colon length by ~25% while Ti_3_C_2_ NSs treatment exhibited a protective effect and completely inhibited the shortening of the colon length (Figure 4C-D). Besides, the severely collapsed structure of colonic tissue, observed in the DSS-induced colitis mice (Figure 4E), significantly improved in histological appearance and regular colonic morphology was observed, demonstrating a great therapeutic outcome for IBD by Ti_3_C_2_ NSs treatment.

To understand the therapeutic mechanism of the *in vivo* IBD therapy and verify ROS elimination by Ti_3_C_2_ NSs, DCFH-DA staining of colon tissue slices was carried out for evaluating the ROS level in the colon after various treatments. The colon from DSS-induced colitis mice showed strong green fluorescence that sharply decreased to the normal level following treatment with Ti_3_C_2_ NSs (Figure 4F, Figure S26). Ti_3_C_2_ NSs aggregation in the colon site was observed by Bio-TEM imaging (Figure 4G), and Ti_3_C_2_ NSs concentration in the colon tissue was maintained at a high level during the therapeutic period (Figure 4H). Furthermore, the colon Ti_3_C_2_ NSs accumulation in colitis mice was higher than in normal mice, confirming that the negatively-charged Ti_3_C_2_ NSs could specifically adsorb to positively charged inflamed colonic tissues via electrostatic interactions (Figure S27).

**Figure 5:**
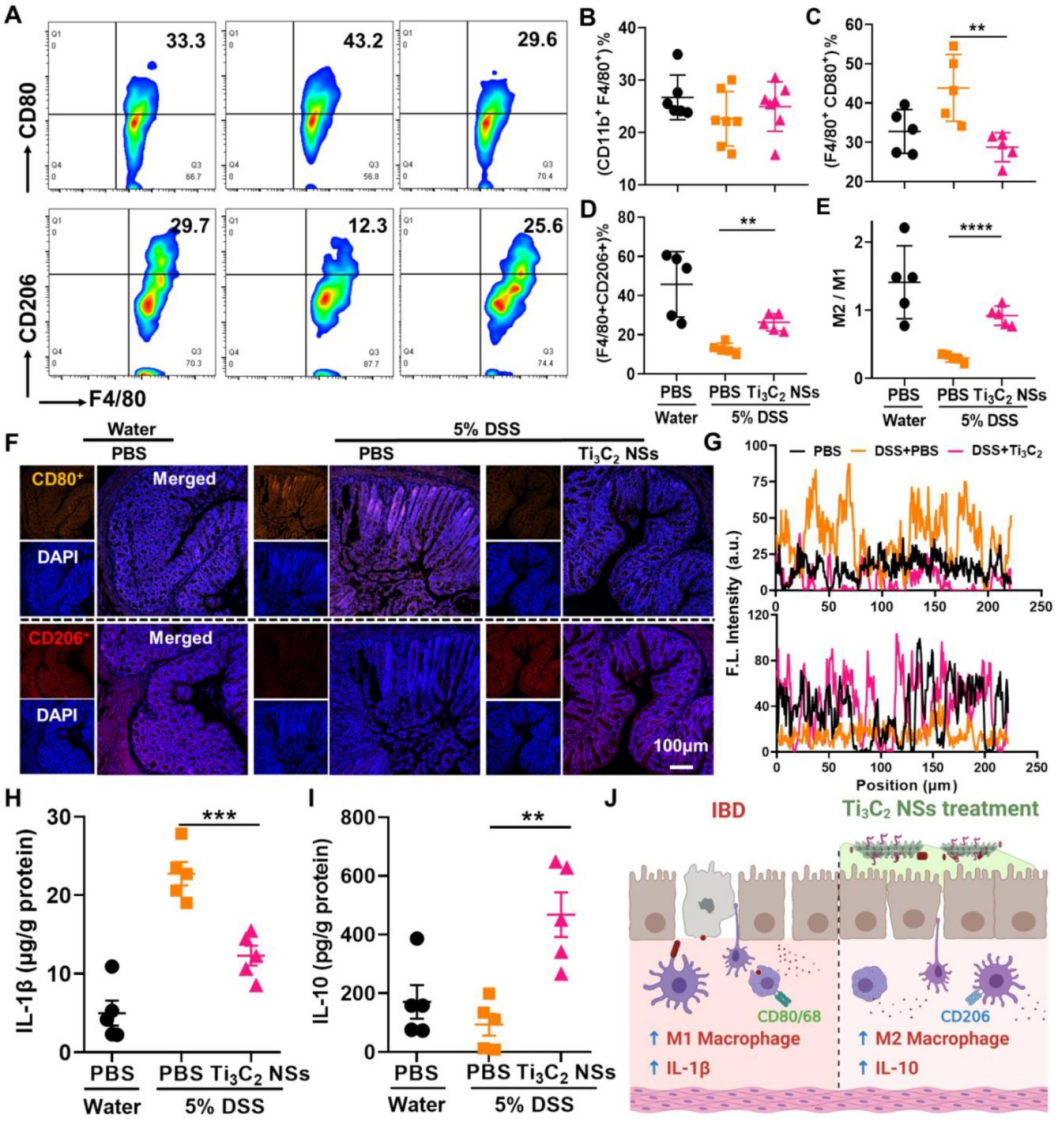
** The colon immune microenvironment variation induced by Ti_3_C_2_ NSs after oral administration.** (**A**) Representative flow cytometry analysis results of M1-phenotype macrophages (CD11b^+^F4/80^+^CD80^+^) and M2-phenotype macrophages (CD11b^+^F4/80^+^CD206^+^) within these colons after different treatments. (**B-E**) The quantitative analysis results of total macrophages (**B**), M1-phenotype macrophages (**C**), M2-phenotype macrophages (**D**), and M2/M1 (**E**). (**F**) Confocal images of colon tissues stained by staining CD80 (M1 phenotype macrophage marker) and CD206 (M2 phenotype macrophage marker) from different groups. (**G**) The statistical analysis of these results in **F**. (**H-I**) The secretion level of IL-1β and IL-10 within the colon tissues from various groups. (**J**) Schematic illustration shows that Ti_3_C_2_ NSs could regulate the colon immune microenvironment to treat IBD. The statistical significance was calculated by two-tailed student's t test. *P < 0.05; **P < 0.01; ***P < 0.005; ****P < 0.001.

Next, variations in the *in vivo* colon microenvironment of IBD mice after the oral administration of Ti_3_C_2_ NSs were investigated. It is well established that the immune response imbalance of the colon causes inflammation and even colitis, and regulating the phenotype of macrophages could alleviate colitis symptoms 37. Therefore, we evaluated the regulation of M1 and M2 macrophage phenotypes in colon tissues following Ti_3_C_2_ NS treatment. Compared with the control group, the total macrophages in the colitis and Ti_3_C_2_ NS-treated colitis groups remained almost unchanged (Figure 5B). However, significantly increased M1-phenotype and decreased M2-phenotype macrophages were observed in DSS-induced colitis while the changes in macrophage phenotypes were reversed by the Ti_3_C_2_ NSs treatment (Figure 5A, 5C-D). Furthermore, the increased ratio of M2 to M1 macrophage infiltration in the colon tissue remarkably alleviated colitis symptoms (Figure 5E). These results were also confirmed by immunofluorescence staining (Figure 5F-G). Similarly, the pro-inflammatory cytokine interleukin-1β (IL-1β) was down-regulated, and the anti-inflammatory cytokine interleukin-10 (IL-10) was up-regulated in the Ti_3_C_2_ NS-treated group (Figure 5H-I). These results indicated that oral administration of Ti_3_C_2_ NSs could efficiently alleviate colitis symptoms by reversing the polarization of the macrophages from the M1 to M2 phenotype and regulating the secretion of various cytokines inside the colon (Figure 5J).

**Figure 6:**
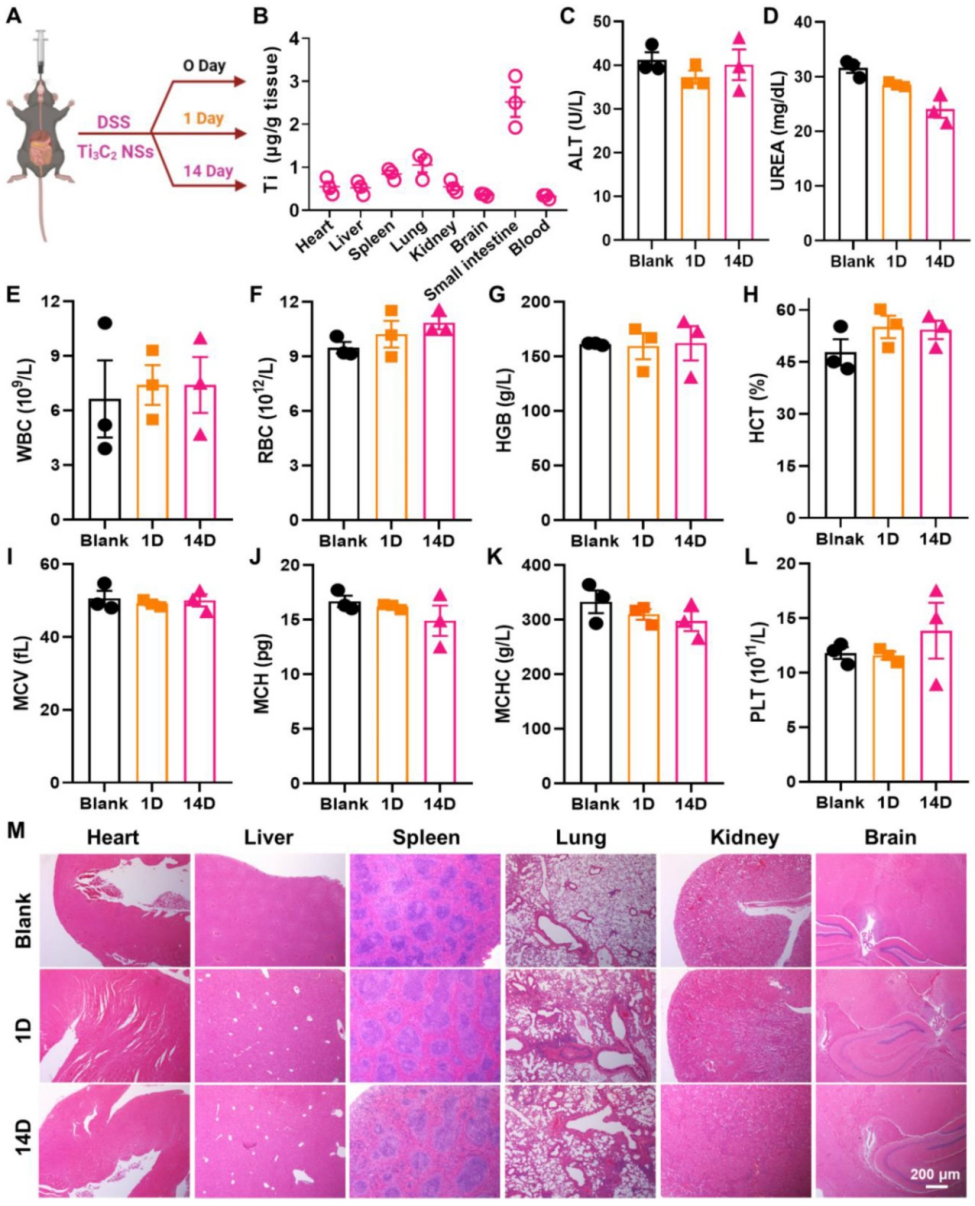
***In vivo* biosafety of Ti_3_C_2_ NSs after oral administration.** (**A**) Scheme of the biosafety assessment of Ti_3_C_2_ NSs after oral administration. (**B**) Biodistribution of Ti_3_C_2_ NSs in mice organs at 7 day. (**C-D**) Blood biochemistry analysis results of these Ti_3_C_2_-treated mice (alanine aminotransferase (ALT, **C**) and urea (**D**)). (**E-L**) Complete blood panel analysis results of above mice (white blood cells (WBC, **E**), red blood cells (RBC, **F**), hemoglobin (HGB, **G**), hematocrit (HCT, **H**), mean corpuscular volume (MCV, **I**), mean corpuscular hemoglobin (MCH, **J**), mean corpuscular hemoglobin concentration (MCHC, **K**), and platelet (PLT, **L**)). (**M**) Microscopy images of H&E-stained mice major organs at different treatment days.

Biosafety is a critical issue for the biological application of nanomaterials. Therefore, the biodistribution of Ti_3_C_2_ NSs in major organs of mice was investigated (Figure 6A). Mice were orally administered Ti_3_C_2_ NSs for 7 consecutive days, and the Ti element level in major organs was measured by ICP-OES following treatment with Ti_3_C_2_ NSs. Notably, apart from the small intestine, no noticeable distribution was observed in other major organs (<1.25 μg/g tissue) (Figure 6B), indicating negligible toxicity of Ti_3_C_2_ NSs. The potential toxicity of Ti_3_C_2_ NSs was further evaluated by blood chemistry, complete blood panel analysis, and histology examination. The blood chemistry parameters and complete blood panel analysis in the orally administered Ti_3_C_2_ NSs group showed no significant difference compared with the control groups (Figure 6C-L). Hematoxylin and eosin (H&E) staining and histology analysis also showed no apparent tissue damage and adverse effect in the Ti_3_C_2_ NS group (Figure 6M). These data showed no significant side effects in mice, indicating a promising potential of orally administered Ti_3_C_2_ NSs for *in vivo* biomedical applications in the future.

**Figure 7:**
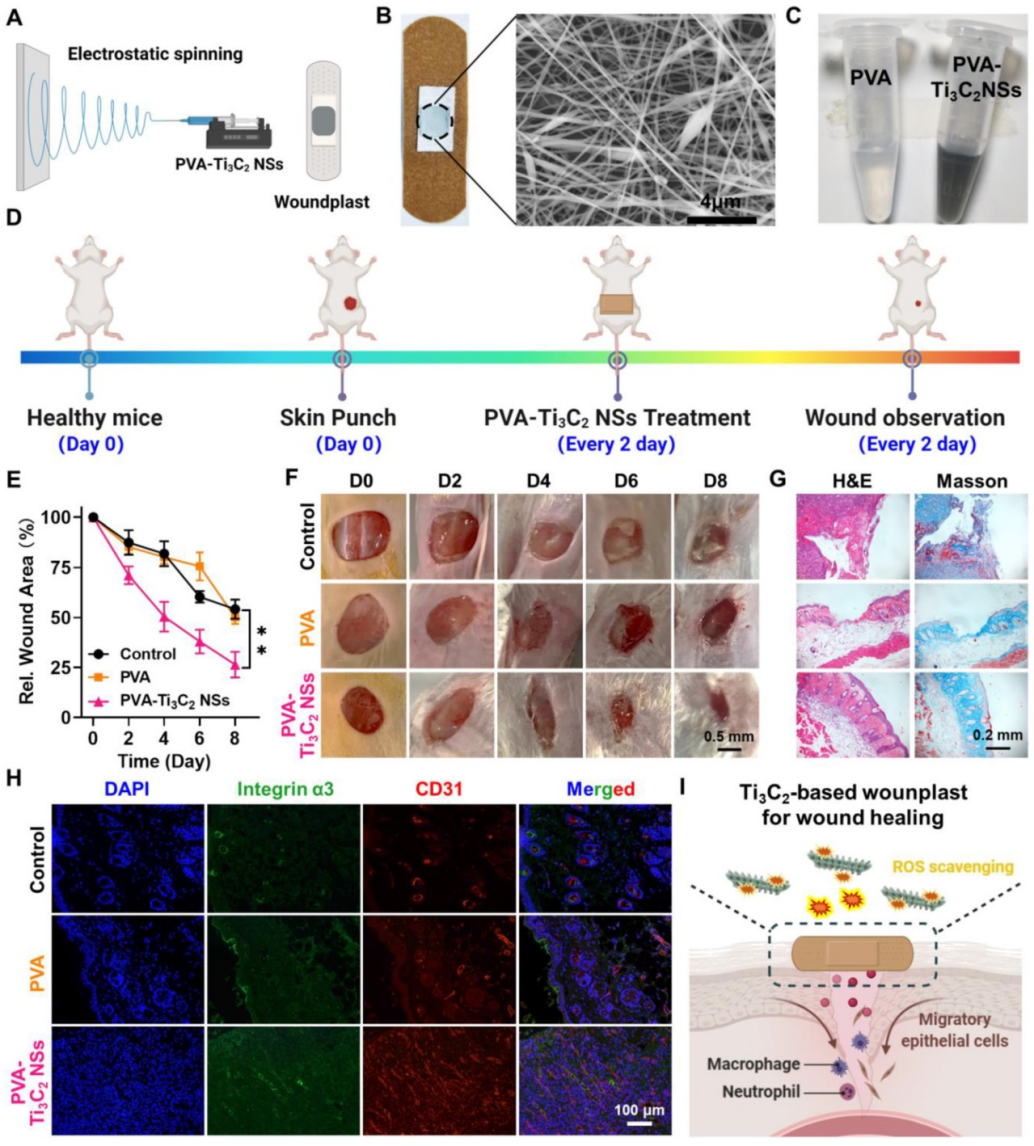
** Skin wound healing on mice treated by Ti_3_C_2_-based woundplast.** (**A**) Schematic illustration shows the preparation process of Ti_3_C_2_ NSs-based woundplast. (**B**) A photograph and its SEM image of our woundplast. (**C**) A photograph of the solutions of PVA or PVA-Ti_3_C_2_ fibers dissolved into PBS buffer. (**D**) Scheme depicts the skin wound healing process of mice treated with Ti_3_C_2_ NSs-based woundplast. (**E**) Relative wound areas after different treatments. (**F**) The images of skin wounds after treatments as indicated. (**G**) Micrographs of H&E- and Masson- stained wounds from various group in **E**. (**H**) The immunofluorescence images of CD31- and integrin α3- stained wound sections. (**I**) Scheme reveals the wound healing mechanism after treatment with Ti_3_C_2_ NSs-based woundplast. The statistical significance was calculated by two-tailed student's t test. *P < 0.05; **P < 0.01; ***P < 0.005; ****P < 0.001.

The excellent *in vivo* ROS-scavenging performance of Ti_3_C_2_ NSs prompted us to investigate its potential application in wound healing. Skin wounds are a frequent occurrence in our daily lives, and the high oxidative stress within the impaired wound largely impedes the wound healing process. Given the role of ROS in wound healing and considering the excellent ROS-scavenging ability of Ti_3_C_2_ NSs, we employed electrospinning technology to prepare Ti_3_C_2_ NS-containing polyvinyl alcohol (PVA) fibers and obtained Ti_3_C_2_-based woundplasts (Figure 7A)^38^. Incorporation of Ti_3_C_2_ NSs into PVA spinning solution yielded Ti_3_C_2_-PVA fibers with black color and uniform Ti_3_C_2_ distribution on their surface (Figure 7B, Figure S28). The Ti_3_C_2_-PVA fibers could be easily dissolved in PBS, enabling efficient ROS scavenging by the released Ti_3_C_2_ NSs (Figure 7C, Figure S29).

Next, we assessed the effect of Ti_3_C_2_-PVA fibers on wound healing by randomly dividing the wound-bearing mice into three groups: (1) Control; (2) PVA-based woundplasts; (3) Ti_3_C_2_-PVA-based woundplasts (4 μg Ti_3_C_2_ NSs per woundplast). These woundplasts were applied on the wounds four times on days 0, 2, 4, and 6 (Figure 7D). The wound areas were monitored every two days in the three groups. Significantly, the wounds in the Ti_3_C_2_-based woundplast-treated group healed quickly and achieved ~73.5% closure on day 8 compared with the control and PVA-treated groups. These results demonstrated effective ROS scavenging by Ti_3_C_2_ NSs to accelerate wound healing (Figure 7E-F). H&E and Masson staining showed that the regenerated wound tissue in the Ti_3_C_2_-based woundplast-treated group was much thicker, and the collagen synthesis was also better than in the control groups (Figure 7G). These data confirmed the significant ability of the Ti_3_C_2_-based woundplast in skin wound healing.

Finally, the skin wounds were examined by immunofluorescence staining using CD31 and integrin α3 (markers of new blood vessels) and collagen. We found increased CD31 and integrin α3 expression in the Ti_3_C_2_-based woundplast group compared with the control groups, indicative of extensive neovascularization in the wounds (Figure 7H). The results demonstrated that the Ti_3_C_2_-based woundplast could efficiently eliminate excess ROS to increase CD31 and the integrin α3 around the wound, promoting skin wound healing and functional vessel formation (Figure 7I).

## Conclusions

In conclusion, the novel antioxidant Ti_3_C_2_ NSs were successfully constructed for specifically targeting to inflammatory colon and effective ROS scavenging via oral administration. Ti_3_C_2_ NSs synthesized via a two-step exfoliation method exhibited excellent long-term stability in acidic SGF. The as-synthesized Ti_3_C_2_ NSs exhibited broad-spectrum ROS scavenging properties that could eliminate excess ROS against oxidative stress-induced cell apoptosis. After oral administration, the negatively-charged Ti_3_C_2_ NSs could specifically target the positively-charged inflamed colon lesions via electrostatic interactions and achieve efficient therapy of DSS-induced colitis. The systematic mechanism demonstrated that Ti_3_C_2_ NSs could significantly inhibit inflammation by decreasing the level of ROS and the secretion of the pro-inflammatory cytokine, increasing the infiltration of M2-phenotype macrophages and the secretion of the anti-inflammatory cytokine, therefore efficiently alleviating the colitis symptoms. Besides, owning to its ROS scavenging ability, Ti_3_C_2_-based woundplast were presented for promoting skin wound healing and functional vessel formation. Overall, the constructed Ti_3_C_2_ NSs with great biosafety and robust ROS eliminating ability could represent a promising orally administered antioxidant for treating IBD or other related inflammatory diseases of the digestive tract.

## Supplementary Material

Supplementary methods and figures.

## References

[1] Baumgart DC, Sandborn WJ (2012). Crohn's disease. The Lancet.

[2] Hindryckx P, Jairath V, D'Haens G (2016). Acute severe ulcerative colitis: from pathophysiology to clinical management. Nat Rev Gastroenterol Hepatol.

[3] de Souza HSP, Fiocchi C (2016). Immunopathogenesis of IBD: current state of the art. Nat Rev Gastroenterol Hepatol.

[4] Bernstein CN, Fried M, Krabshuis JH, Cohen H, Eliakim R, Fedail S (2010). World gastroenterology organization practice guidelines for the diagnosis and management of IBD in 2010. Inflamm Bowel Dis.

[5] Hoivik ML, Moum B, Solberg IC, Cvancarova M, Hoie O, Vatn MH (2012). Health-related quality of life in patients with ulcerative colitis after a 10-year disease course: results from the IBSEN study. Inflamm Bowel Dis.

[6] Sela-Passwell N, Kikkeri R, Dym O, Rozenberg H, Margalit R, Arad-Yellin R (2012). Antibodies targeting the catalytic zinc complex of activated matrix metalloproteinases show therapeutic potential. Nat Med.

[7] Wilson DS, Dalmasso G, Wang L, Sitaraman SV, Merlin D, Murthy N (2010). Orally delivered thioketal nanoparticles loaded with TNF-α-siRNA target inflammation and inhibit gene expression in the intestines. Nat Mater.

[8] Dickson I (2017). Ustekinumab therapy for Crohn's disease. Nat Rev Gastroenterol Hepatol.

[9] Lautenschläger C, Schmidt C, Fischer D, Stallmach A (2014). Drug delivery strategies in the therapy of inflammatory bowel disease. Adv Drug Delivery Rev.

[10] Chung CH, Jung W, Keum H, Kim TW, Jon S (2020). Nanoparticles derived from the natural antioxidant rosmarinic acid ameliorate acute inflammatory bowel disease. ACS Nano.

[11] Zhang S, Ermann J, Succi Marc D, Zhou A, Hamilton Matthew J, Cao B (2015). An inflammation-targeting hydrogel for local drug delivery in inflammatory bowel disease. Sci Transl Med.

[12] Dickinson BC, Chang CJ (2011). Chemistry and biology of reactive oxygen species in signaling or stress responses. Nat Chem Biol.

[13] Huang LJ, Mao XT, Li YY, Liu DD, Fan KQ, Liu RB (2021). Multiomics analyses reveal a critical role of selenium in controlling T cell differentiation in Crohn's disease. Immunity.

[14] Canny G, Levy O, Furuta GT, Narravula-Alipati S, Sisson RB, Serhan CN (2002). Lipid mediator-induced expression of bactericidal/permeability-increasing protein (BPI) in human mucosal epithelia. Proc Natl Acad Sci.

[15] Zhang S, Langer R, Traverso G (2017). Nanoparticulate drug delivery systems targeting inflammation for treatment of inflammatory bowel disease. Nano Today.

[16] Han W, Mercenier A, Ait-Belgnaoui A, Pavan S, Lamine F, van Swam II (2006). Improvement of an experimental colitis in rats by lactic acid bacteria producing superoxide dismutase. Inflamm Bowel Dis.

[17] Zhao S, Li Y, Liu Q, Li S, Cheng Y, Cheng C (2020). An Orally Administered CeO_2_@montmorillonite nanozyme targets inflammation for inflammatory bowel disease therapy. Adv Funct Mater.

[18] Vong LB, Tomita T, Yoshitomi T, Matsui H, Nagasaki Y (2012). An orally administered redox nanoparticle that accumulates in the colonic mucosa and reduces colitis in mice. Gastroenterology.

[19] Vong LB, Yoshitomi T, Matsui H, Nagasaki Y (2015). Development of an oral nanotherapeutics using redox nanoparticles for treatment of colitis-associated colon cancer. Biomaterials.

[20] Zhang Q, Tao H, Lin Y, Hu Y, An H, Zhang D (2016). A superoxide dismutase/catalase mimetic nanomedicine for targeted therapy of inflammatory bowel disease. Biomaterials.

[21] Feng W, Han X, Hu H, Chang M, Ding L, Xiang H (2021). 2D vanadium carbide MXenzyme to alleviate ROS-mediated inflammatory and neurodegenerative diseases. Nat Commun.

[22] Chu B, Wang A, Cheng L, Chen R, Shi H, Song B (2021). Ex vivo and *in vivo* fluorescence detection and imaging of adenosine triphosphate. J Nanobiotechnol.

[23] Zhao X, Wang LY, Li JM, Peng LM, Tang CY, Zha XJ (2021). Redox-mediated artificial non-enzymatic antioxidant MXene nanoplatforms for acute kidney injury alleviation. Adv Sci.

[24] Naguib M, Kurtoglu M, Presser V, Lu J, Niu J, Heon M (2011). Two-dimensional nanocrystals produced by exfoliation of Ti_3_AlC_2_. Adv Mater.

[25] Szuplewska A, Rozmysłowska-Wojciechowska A, Poźniak S, Wojciechowski T, Birowska M, Popielski M (2019). Multilayered stable 2D nano-sheets of Ti_2_NTx MXene: synthesis, characterization, and anticancer activity. J Nanobiotechnol.

[26] Tang W, Dong Z, Zhang R, Yi X, Yang K, Jin M (2019). Multifunctional two-dimensional core-shell MXene@gold nanocomposites for enhanced photo-radio combined therapy in the second biological window. ACS Nano.

[27] Low J, Zhang L, Tong T, Shen B, Yu J (2018). TiO_2_/MXene Ti_3_C_2_ composite with excellent photocatalytic CO_2_ reduction activity. J Catal.

[28] Lotfi R, Naguib M, Yilmaz DE, Nanda J, van Duin ACT (2018). A comparative study on the oxidation of two-dimensional Ti_3_C_2_ MXene structures in different environments. J Mater Chem A.

[29] Xu Y, Wang S, Yang J, Han B, Nie R, Wang J (2018). In-situ grown nanocrystal TiO_2_ on 2D Ti_3_C_2_ nanosheets for artificial photosynthesis of chemical fuels. Nano Energy.

[30] Weng Q, Sun H, Fang C, Xia F, Liao H, Lee J (2021). Catalytic activity tunable ceria nanoparticles prevent chemotherapy-induced acute kidney injury without interference with chemotherapeutics. Nat Commun.

[31] Wang T, Li Y, Cornel EJ, Li C, Du J (2021). Combined antioxidant-antibiotic treatment for effectively healing infected diabetic wounds based on polymer vesicles. ACS Nano.

[32] Ni D, Jiang D, Kutyreff CJ, Lai J, Yan Y, Barnhart TE (2018). Molybdenum-based nanoclusters act as antioxidants and ameliorate acute kidney injury in mice. Nat Commun.

[33] Yim D, Lee D-E, So Y, Choi C, Son W, Jang K (2020). Sustainable nanosheet antioxidants for sepsis therapy via scavenging intracellular reactive oxygen and nitrogen species. ACS Nano.

[34] Dehghani MA, Shakiba Maram N, Moghimipour E, Khorsandi L, Atefi khah M, Mahdavinia M (2020). Protective effect of gallic acid and gallic acid-loaded Eudragit-RS 100 nanoparticles on cisplatin-induced mitochondrial dysfunction and inflammation in rat kidney. BBA-MOL Basis Dis.

[35] Lee B-C, Lee Jin Y, Kim J, Yoo Je M, Kang I, Kim J-J Graphene quantum dots as anti-inflammatory therapy for colitis. Sci Adv. 6: eaaz2630.

[36] Shen Q, Huang Z, Yao J, Jin Y (2021). Extracellular vesicles-mediated interaction within intestinal microenvironment in inflammatory bowel disease. J Adv Res.

[37] Lee Y, Sugihara K, Gillilland MG, Jon S, Kamada N, Moon JJ (2020). Hyaluronic acid-bilirubin nanomedicine for targeted modulation of dysregulated intestinal barrier, microbiome and immune responses in colitis. Nat Mat.

[38] Müller GFJ, Stürzel M, Mülhaupt R (2014). Core/shell and hollow ultra gigh molecular weight polyethylene nanofibers and nanoporous polyethylene prepared by mesoscopic shape replication catalysis. Adv Funct Mater.

