# Supplementary material for: Orally administered titanium carbide nanosheets as anti-inflammatory therapy for colitis
Source: Theranostics. 2022 May 9;12(8):3834–46. doi: 10.7150/thno.70668 (PMC9131285; doi:10.7150/thno.70668)
Supplement: Supplementary file 1 — Supplementary methods and figures. [file thnov12p3834s1.pdf]

Orally administered titanium carbide nanosheets as anti-inflammatory therapy for colitis

Linqian Hou[1], Fei Gong[1]*, Bo Liu[1], Xiaoyuan Yang[1], Linfu Chen[1], Guangqiang Li[2], Yuehan Gong[1], Chao Liang[3], Nailin Yang[1], Xian Shen[3]*, Zhuang Liu[1], and Liang Cheng[1]*

[1]Institute of Functional Nano & Soft Materials (FUNSOM), Jiangsu Key Laboratory for Carbon-Based Functional Materials & Devices, Soochow University, Suzhou 215123, China

[2]College of Science, State Key Laboratory of Agricultural Microbiology, Huazhong Agricultural University, Wuhan, 430070, China

[3]Department of Gastrointestinal Surgery, The Second Affiliated Hospital, Wenzhou Medical University, Wenzhou, Zhejiang Province, China

E-mails: lcheng2@suda.edu.cn; gongfei@suda.edu.cn; shenxian1120@126.com

Experimental Sections

Materials and chemicals

The Ti_3AlC_2 powder (~38 μm) and dextran sulfate sodium salt (DSS, 40 kDa) were purchased from Beijing Hwrkchemical Co., Ltd. Hydrofluoric acid (HF) (40%) was obtained from the Shanghai Macklin Biochemical Co., Ltd. Polyvinyl pyrrolidone (PVP, MW 10k), 3,3,5,5-tetramethylbenzidine (TMB), 1,1-diphenyl-2-picrylhydrazyl (DPPH), and 2,2-azino-bis(3-ethylbenzothiazoline-6-sulfonic acid) (ABTS) were obtained from Sigma-Aldrich. Tetra-n-propylammonium hydroxide (TPAOH, 25%) was purchased from Shanghai Shaoyuan Co. Ltd. All other agents were of analytical grade and used without further purification.

Synthesis of Ti_3C_2 NSs

The Ti_3C_2 nanosheets (NSs) were synthesized via two-step exfoliation strategy, which was based on HF etching and TPAOH intercalation of Ti_3AlC_2 powder [1]. In brief, 20 mL HF (20%) was added slowly into 1 g Ti_3AlC_2 powder under stirring to remove the middle Al layer. After reaction for 4 h, the obtained Ti_3C_2 powder was centrifuged and washed repeatedly with water to remove the excess HF. Subsequently, the Ti_3C_2 powder was exfoliated in 20 mL TMPOH (~25%) and kept under stirring overnight. After collecting by centrifugation and washing with water and ethanol for three times, the final Ti_3C_2 NSs was obtained.

Modification of Ti_3C_2 NSs

The synthesized Ti_3C_2 NSs were modified by polyvinylpyrrolidone (PVP, MW 10k). Briefly, 50 mg Ti_3C_2 NSs and 250 mg PVP were dissolved in 20 ml H_2O and stirred for 4 h. After dialysis (8-14.8 kDa), the PVP modified Ti_3C_2 NSs were dispersed in water and stored in a -20 ^{o}C for future use (concentration, 1 mg/mL).

Characterization

Scanning electron microscopy (SEM) imaging was conducted by a ZEISS G500 SEM. Transmission electron microscopy (TEM) imaging and elemental mapping were performed

by a FEI Tecnai F20 TEM. Phase purity of the Ti_3AlC_2 powder and its structural transformation were examined by powder X-ray diffraction (XRD) using a PANalytical X-ray diffractometer equipped with CuKα radiation (λ=0.15406 nm). The surface states of Ti_3C_2 NSs were characterized on the PHI Quantera SXM X-ray photoelectron spectrometer (XPS) with an Al Ka monochromator source. Atomic force microscopy (AFM) measurement was performed using a Veeco AFM. UV-vis-NIR absorbance spectrum was measured by PerkinElmer Lambda 750 UV-vis-NIR spectrophotometer. The concentration of Ti_3C_2 NSs was calculated by Ti as measured by inductively coupled plasma atomic emission spectrometer (ICP-OES).

ROS scavenging assays

H_2O_2 assays: Different concentrations of H_2O_2 (0.6, 1.2, 2.5, and 5 mM) mixed with Ti_3C_2 NSs (25 μg/mL) for 30 min reaction, then the absorption of Ti_3C_2 NSs was measured to determine the surplus of them.

ABTS+• radical assay: Initially, the ABTS+• radical were prepared by mixing ABTS (7 mM) and potassium persulfate ($K_2S_2O_8$, 2.45 mM) for fully reacted overnight at 4 ^oC. Then, Ti_3C_2 NSs with proper concentrations (0.5, 1, 2, and 4 μg/mL) incubated with ABTS+• for 10 min, and their absorption at 734 nm were recorded.

DPPH• radical assay: Different concentrations of Ti_3C_2 NSs (0.5, 1, 2, and 4 μg/mL) mixed with DPPH• (60 μM) in ethanol for 5 min, then their absorption at 517 nm was measured.

TMB assay: Since hydroxyl radical (HO•) produced by the Fenton reaction could oxidize TMB into *ox*TMB, the absorption of *ox*TMB could reflect the HO• scavenging ability of Ti_3C_2 NSs. The Fenton agents (Fe^{2+}: 10 μM; H_2O_2: 50 μM) mixed with TMB (0.3 mM) and then incubated with Ti_3C_2 NSs (2, 4, and 8 μg/mL). After reaction for 10 min, the absorption of *ox*TMB at 654 nm was monitored by UV-vis-NIR spectrophotometer.

TEMPO assay: TEMPO (20 μM) as intrinsic free radicals were examined by the electron spin resonance (ESR) spectra w/wo addition of Ti_3C_2 NSs. The characteristic peaks

intensity revealed the concentration of TEMPO and further verified the antioxidative properties of Ti_3C_2 NSs.

Antioxidant efficiency and mechanism

According to the previous literatures, we selected four materials like cerium dioxide (CeO_2) [2-4], Mo-based polyoxometalate (POM) cluster [5], tungsten sulfide (WS_2) [6], and gallic acid (GA) [7] to compare ROS scavenging performance of them with Ti_3C_2 NSs. And ABTS+• radical were employed to evaluate the ROS scavenging ability of these materials. In addition, the fresh Ti_3C_2 NSs, lightly oxidized Ti_3C_2 NSs, and heavily oxidized Ti_3C_2 NSs were characterized by XPS analysis and Raman spectrum to reveal the microstructure and the antioxidant mechanism of our Ti_3C_2 NSs.

Cellular experiments

Human colonic epithelial cells (HT29) and mouse macrophage cells (RAW 264.7) were cultured in a normal RPMI-1640 medium or DMEM supplemented with 10% fetal bovine serum (FBS) and 1% penicillin/streptomycin at 37 ^{o}C under 5% CO_2 atmosphere, respectively.

In vitro cytotoxicity test: HT29 cells were seeded in 96-well plates were incubated with Ti_3C_2 NSs at different concentrations (10, 20, 40, 60, 80, and 100 μg/mL) for 12 h, then the relative cell viabilities were detected by the standard methyl thiazolyl tetrazolium (MTT) assay.

Intracellular ROS scavenging assays: HT29 cells were seeded in 96-well plates and then co-incubated with Ti_3C_2 NSs (80 μg/mL) and H_2O_2 (1 or 2 mM) for 10 h. The standard MTT assay was conducted to test the relative cell viabilities. In addition, these above-treated HT29 cells were also stained with Calcein AM (AM, green, live cell) and propidium iodide (PI, red, dead cell) to determine the cell protection by Ti_3C_2 NSs. Finally, the HT29 cells after co-incubation with Ti_3C_2 NSs (80 μg/mL) and H_2O_2 (200 μM) were stained with 2,7-dichlorofluorescein diacetate (DCFH-DA, 20 μM) to quantify the intracellular ROS

levels. All the images were acquired by a confocal laser scanning microscope (CLSM, Zeiss Axio-Imager LSM-800).

Macrophages polarization assays: RAW 264.7 were seeded and then incubated with lipopolysaccharide (LPS) or LPS/Ti_3C_2 NSs (40 μg/mL). These above RAW 264.7 were stained by CD80 marker and then analyzed by CLSM and flow cytometric examination to evaluate the polarization of macrophages and the inhibiting effect of Ti_3C_2 NSs for LPS-induced macrophages polarization.

Animal model

Six-week-old female C57BL/6 and BALB/c mice (18 ± 2 g) were purchased from Nanjing Sikerui Biological Technology Co. Ltd, and all animal experiments were carried out under the permission of Soochow University Laboratory Animal Center.

DSS-induced colitis model

Female C57BL/6 mice were grouped randomly and acclimatized for one week. Subsequently, these mice were given 5% DSS supplemented in the drinking water for 5 days to induce inflammatory bowel disease (IBD), then returned to normal water.

IBD treatment by Ti_3C_2 NSs

The healthy mice in the control group were provided with normal water during the whole time. And the DSS-induced IBD mice were orally administered by Ti_3C_2 NSs (45 mg/kg) or PBS on the planned days. The body weights of these mice were monitored daily during the experimental period. On the last day over the experiment, the mice were sacrificed and their colons were collected. The colon lengths of these colons were collected, and then they were gently rinsed with physiological saline to remove the intestinal contents. Finally, two sections of the distal tissue with a length of 0.5 cm were taken for histological evaluation and fluorescence analysis. The remaining colon tissues were used for flow cytometric and cytokine analysis.

Histopathological evaluation

Histological analysis of colon tissue sections stained with hematoxylin and eosin (H&E). In brief, the collected colon tissues were fixed in 4% paraformaldehyde and then embedded in paraffin. The sections of distal colon tissues (5 μm) were made and stained by H&E for analysis.

Colon microenvironment evaluation

The collected colon tissues were placed in PBS buffer, and the intestinal contents were gently washed. Subsequently, these colon tissues were homogenized in FACS buffer, digested in digestive enzyme, and filtrated through nylon gauze to obtain single-cell suspensions. After stained with corresponding fluorescent-labeled antibodies (macrophages: CD11b-PE, F4/80-FITC, CD80-APC, CD206-APC), these cells were analyzed by the flow cytometric analysis.

For immunofluorescence evaluation, the collected colon tissues fixed with tissue freezing medium were sliced. The tissue sections were stained with anti-CD80 or anti-CD206 antibody, following by stained with the corresponding fluorescence-labeled secondary antibodies and DAPI (cell nuclear dye). The immunofluorescence images were conducted by CLSM (Zeiss Axio-Imager LSM-800). The concentrations of cytokines like IL-1β and IL-10 in these colon tissues were determined by enzyme-linked immunosorbent assay (ELISA).

Biosafety of Ti_3C_2 NSs after oral administration

The DSS-induced IBD mice were orally administered by Ti_3C_2 NSs (45 mg/kg) on predetermined days. As the protocol revealed, these mice were scarified at day 0, 1, and 14 after treatment. Then, the blood and major organs were collected to analyze and evaluate the biosafety of the synthesized Ti_3C_2 NSs.

Preparation of Ti_3C_2-based woundplast by electrospinning

Ti_3C_2 NSs with appropriate concentration was mixed with pre-prepared PVA solution to obtain Ti_3C_2-PVA spinning solution. Afterwards, the spinning solution was injected into the syringe with a 20-gauge needle (inner diameter 0.6 mm, outer diameter 0.9 mm) for

electrospinning to prepare Ti_3C_2 NSs-based woundplast (4 μg Ti_3C_2 per piece). The parameters of electrospinning were as follow: voltage, 15 kV; distance between needle tip and collector, 12.0 cm; flow rate, 0.5 mL/h.

Wound closure accelerated by Ti_3C_2 NSs-based woundplast

Healthy six-week-old female BALB/c mice were employed as experimental subjects. Firstly, the back hair of the mice was shaved and disinfected, and then two full-thickness skin wounds in 8 mm diameter were made on the back of each mouse. These mice bearing wounds were randomly divided into three groups (six wounds per group): (1) Control; (2) PVA-based woundplast; (3) Ti_3C_2 NSs-based woundplast. These treatments were repeated every two days and totally conducted four times. After various treatments, these wounds were monitored, measured, and analyzed by Image J software. On the last day of the treatment (day 8), these wounds were excised and stained by H&E and Masson to evaluate the wound healing by Ti_3C_2 NSs-based woundplast. Lastly, these wounds also were stained with anti-CD31 and anti-integrin α3 antibody to reveal the generation of newborn blood vessels and collagen around the wounds.

Statistical Analysis

All the results in this work were presented as mean values ± SD. Statistical analysis was conducted via GraphPad Prism software 6. Two-tailed Student's t-test was used for two group comparison and One-way analysis of variance (ANOVA) with a Tukey post-hoc test was used for multiple comparisons. Statistical differences were calculated with two-tailed student's t-test, $^*p < 0.05$, $^{**}p < 0.01$, and $^{***}p < 0.001$.

References

1. Naguib, M.; Kurtoglu, M.; Presser, V.; Lu, J.; Niu, J.; Heon, M.; Hultman, L.; Gogotsi, Y.; Barsoum, M. W., Two-dimensional nanocrystals produced by exfoliation of Ti3AlC2. *Adv. Mater.* **2011,** *23* (37), 4248-4253.
2. Zhao, S.; Li, Y.; Liu, Q.; Li, S.; Cheng, Y.; Cheng, C.; Sun, Z.; Du, Y.; Butch, C. J.; Wei, H., An Orally Administered CeO2@Montmorillonite Nanozyme Targets Inflammation for Inflammatory Bowel Disease Therapy. *Adv. Funct. Mater.* **2020,** *30* (45), 2004692.

3. Weng, Q.; Sun, H.; Fang, C.; Xia, F.; Liao, H.; Lee, J.; Wang, J.; Xie, A.; Ren, J.; Guo, X.; Li, F.; Yang, B.; Ling, D., Catalytic activity tunable ceria nanoparticles prevent chemotherapy-induced acute kidney injury without interference with chemotherapeutics. *Nat. Commun.* **2021,** *12* (1), 1436.
4. Wang, T.; Li, Y.; Cornel, E. J.; Li, C.; Du, J., Combined Antioxidant–Antibiotic Treatment for Effectively Healing Infected Diabetic Wounds Based on Polymer Vesicles. *ACS Nano* **2021,** *15* (5), 9027-9038.
5. Ni, D.; Jiang, D.; Kutyreff, C. J.; Lai, J.; Yan, Y.; Barnhart, T. E.; Yu, B.; Im, H.-J.; Kang, L.; Cho, S. Y.; Liu, Z.; Huang, P.; Engle, J. W.; Cai, W., Molybdenum-based nanoclusters act as antioxidants and ameliorate acute kidney injury in mice. *Nat. Commun.* **2018,** *9* (1), 5421.
6. Yim, D.; Lee, D.-E.; So, Y.; Choi, C.; Son, W.; Jang, K.; Yang, C.-S.; Kim, J.-H., Sustainable Nanosheet Antioxidants for Sepsis Therapy via Scavenging Intracellular Reactive Oxygen and Nitrogen Species. *ACS Nano* **2020,** *14* (8), 10324-10336.
7. Dehghani, M. A.; Shakiba Maram, N.; Moghimipour, E.; Khorsandi, L.; Atefi khah, M.; Mahdavinia, M., Protective effect of gallic acid and gallic acid-loaded Eudragit-RS 100 nanoparticles on cisplatin-induced mitochondrial dysfunction and inflammation in rat kidney. *BBA-MOL Basis. Dis.* **2020,** *1866* (12), 165911.

Supporting Figures

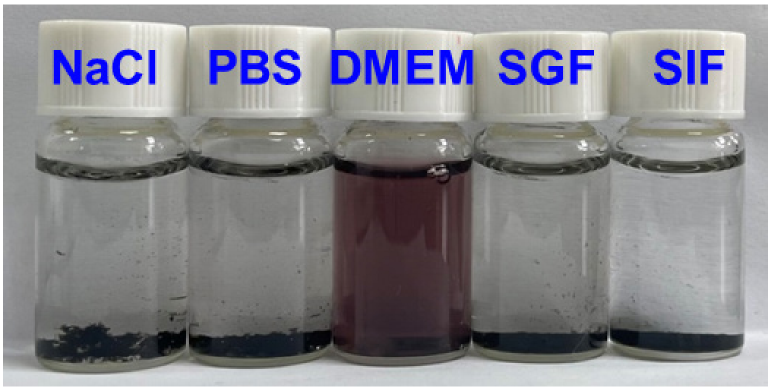

Figure S1. The stability of the Ti_3C_2 NSs in various buffer solutions, including 0.9%NaCl, PBS, DMEM, SGF, and SIF.

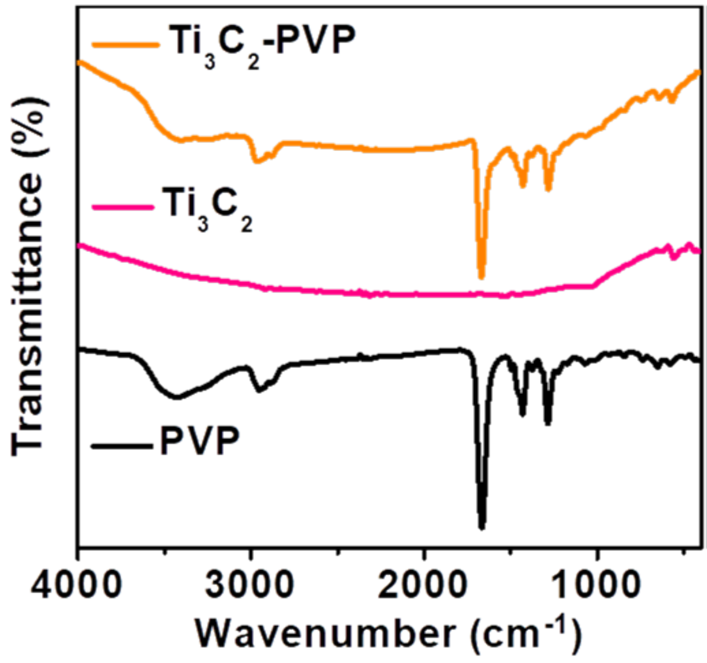

Figure S2. FTIR spectra of Ti_3C_2, Ti_3C_2-PVP, and PVP samples.

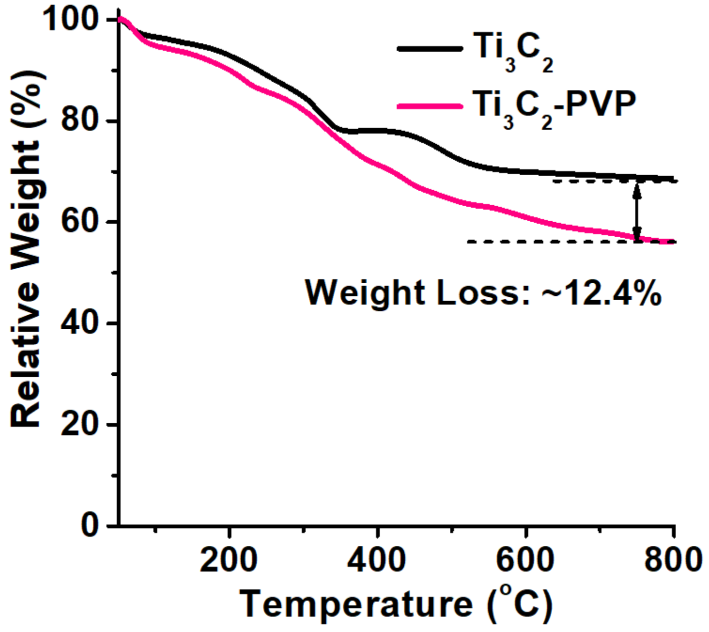

Figure S3. TGA curves of Ti_3C_2 and Ti_3C_2-PVP samples.

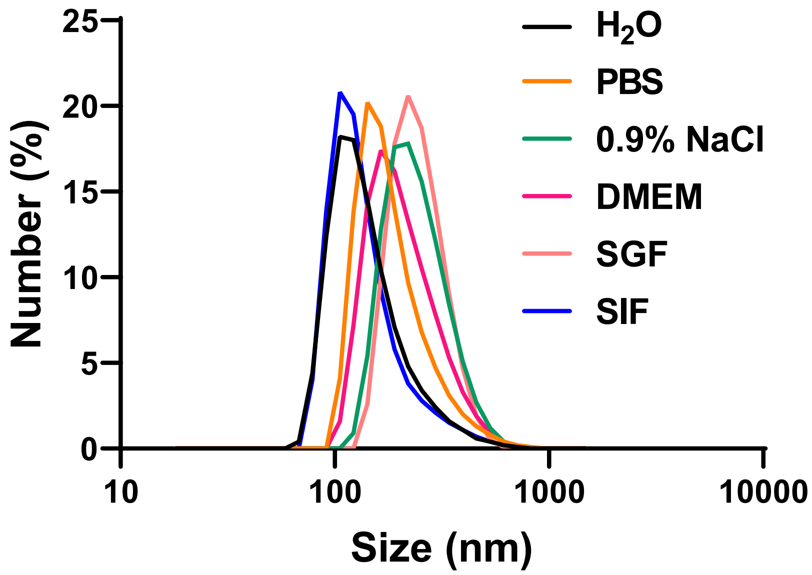

Figure S4. The dynamic light scattering (DLS) of Ti_3C_2 NSs in various buffers including H_2O, PBS, 0.9% NaCl, DMEM, SGF, and SIF.

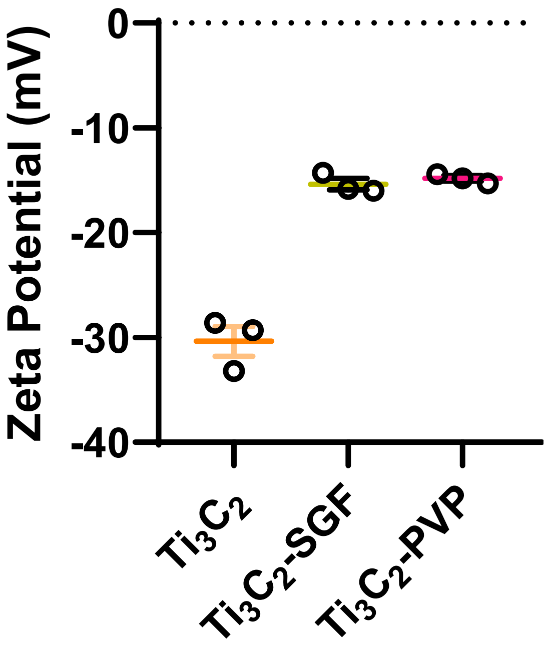

Figure S5. Zeta potentials of Ti_3C_2 NSs, Ti_3C_2-PVP and SGF-treated Ti_3C_2 NSs.

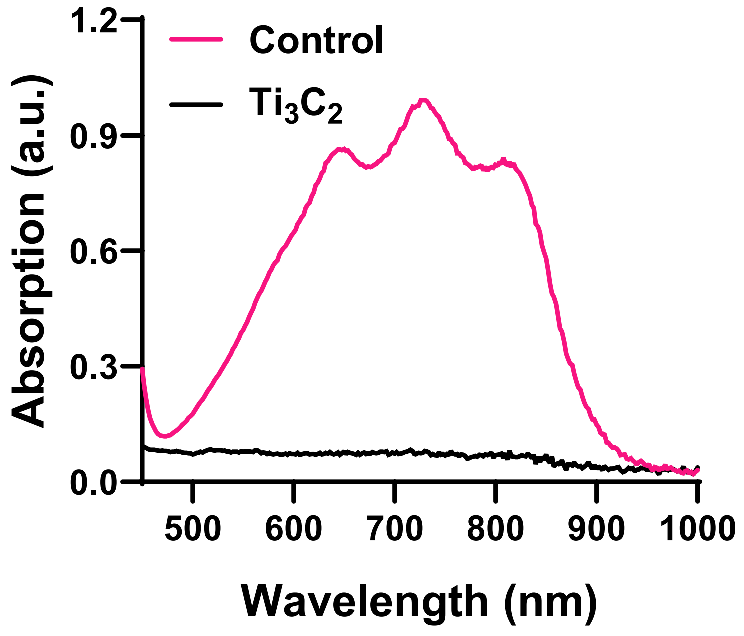

Figure S6. UV-vis-NIR absorbance spectra of ABTS+• radical after incubation with SGF-treated Ti_3C_2 NSs for 1 min (dose: 2 μg/mL).

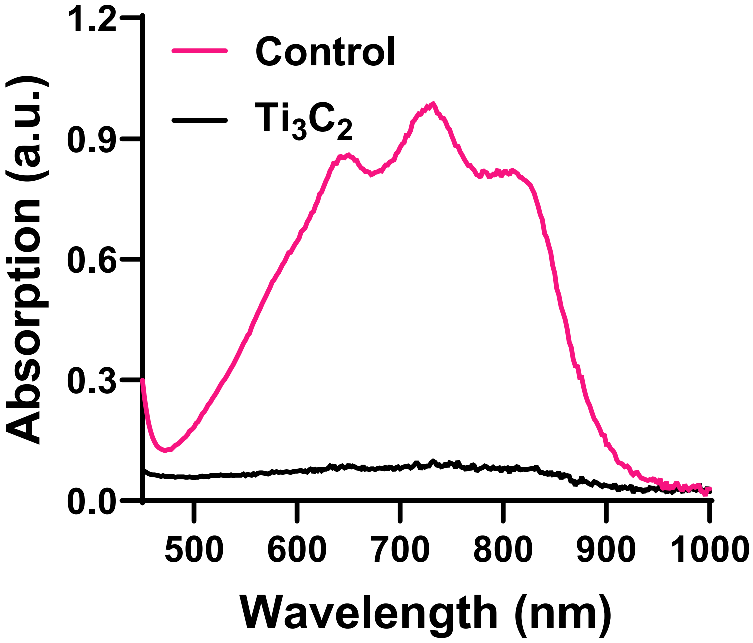

Figure S7. UV-vis absorbance spectra of ABTS+• radical after incubation with Ti_3C_2 NSs in SGF buffers.

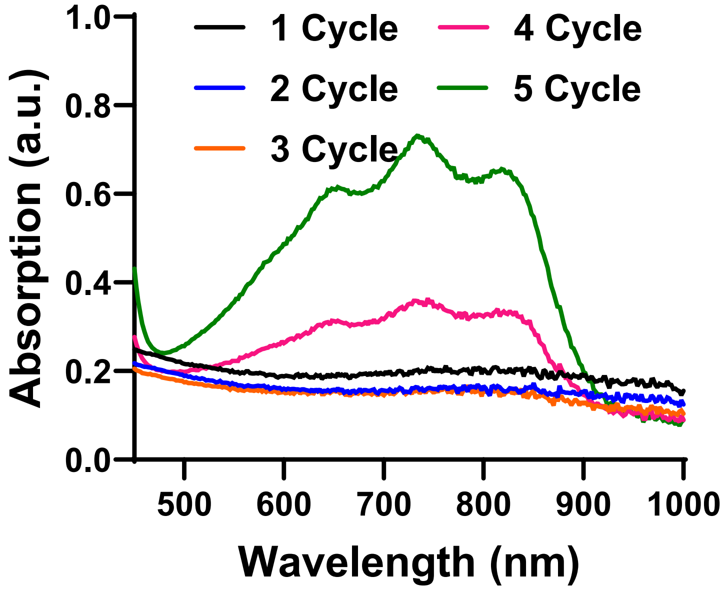

Figure S8. The investigation of ROS scavenging performance by Ti_3C_2 NSs for five cycle times.

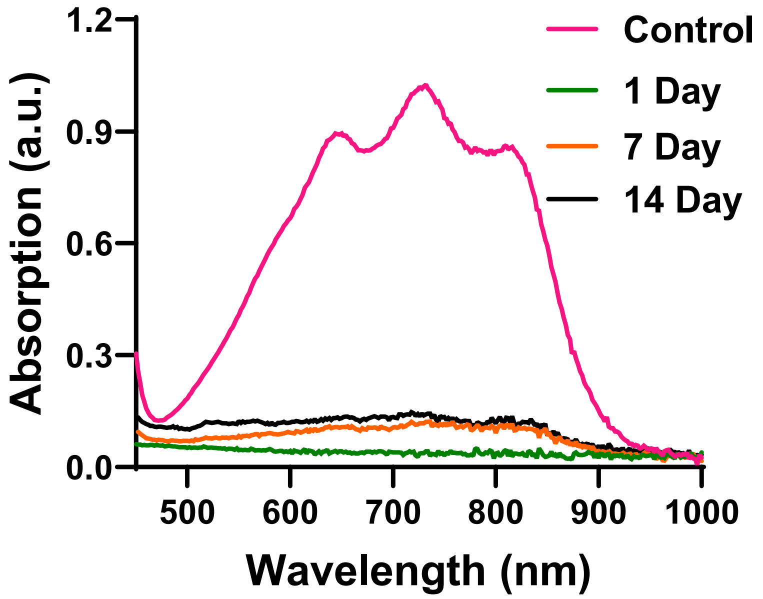

Figure S9. UV-vis absorbance spectra of ABTS+• radical after incubation with different Ti_3C_2 NSs samples.

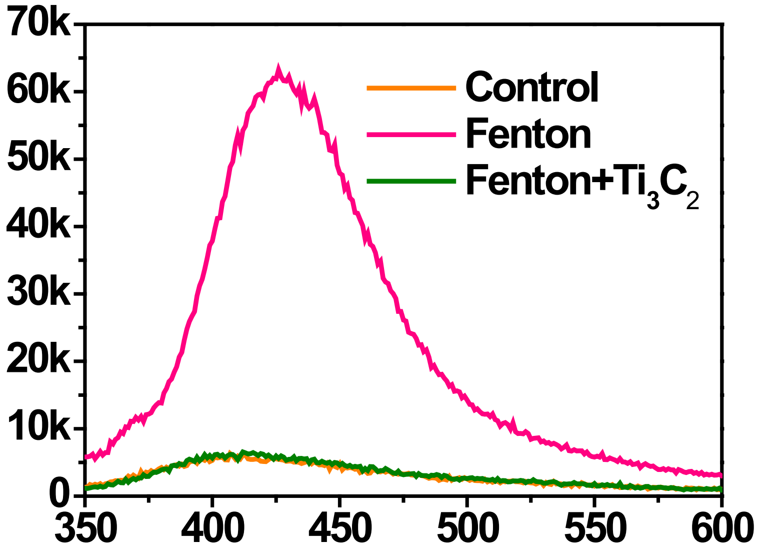

Figure S10. Fluorescence spectra obtained from different reaction groups as indicated.

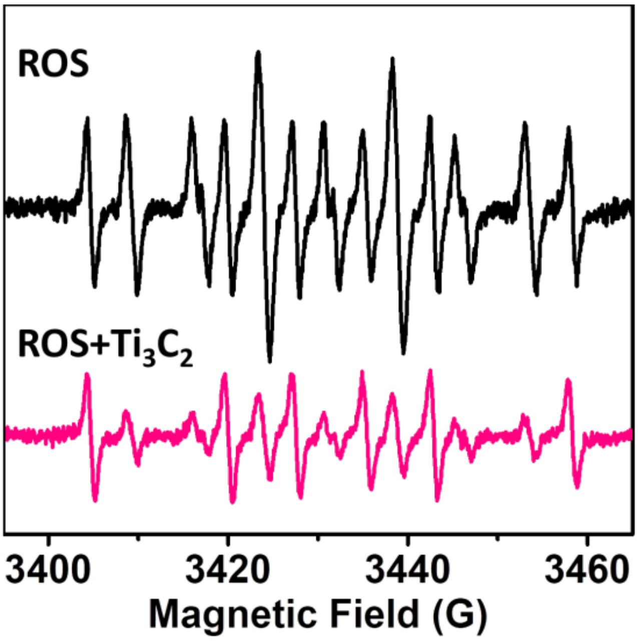

Figure S11. ROS scavenging ability of Ti_3C_2 NSs determined by ESR spectra.

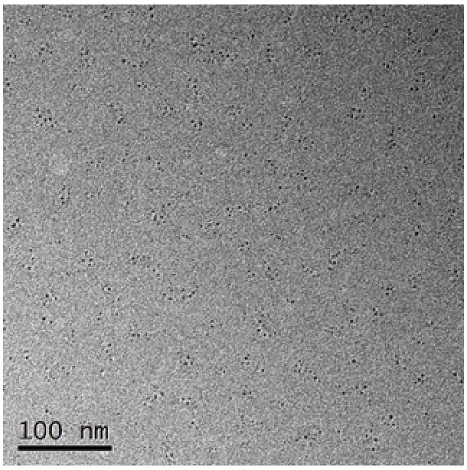

Figure S12. TEM image of Ti_3C_2 NSs after reaction with ROS.

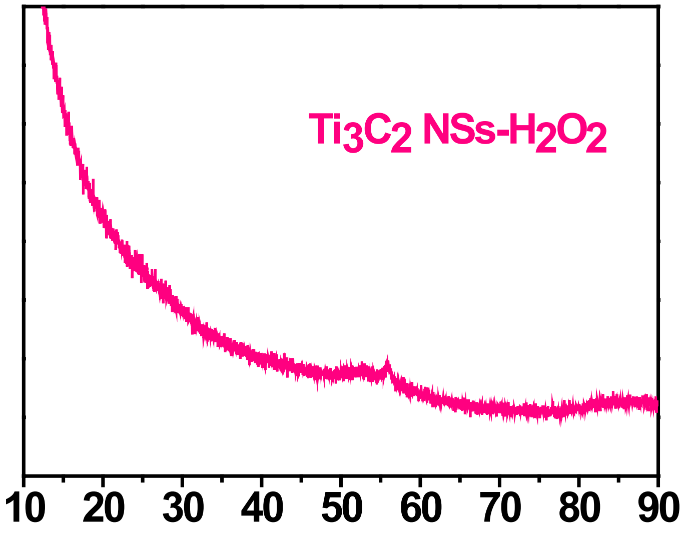

Figure S13. XRD spectrum of Ti_3C_2 NSs after reaction with ROS.

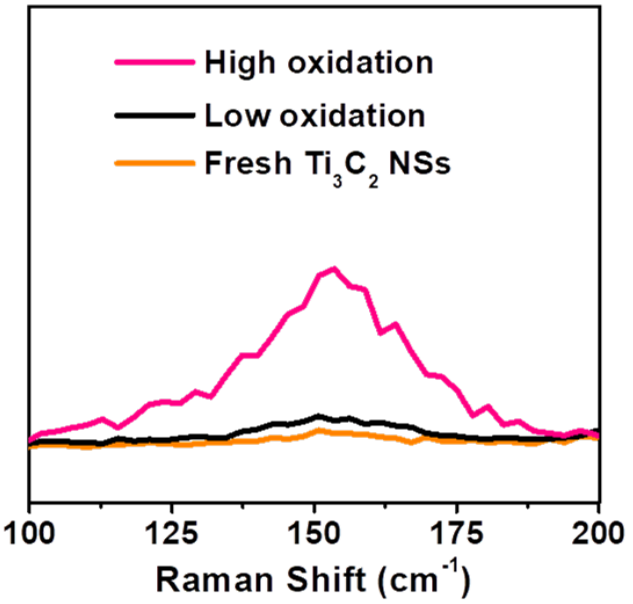

Figure S14. Raman spectra of Ti_3C_2 NSs with different degree of oxidation.

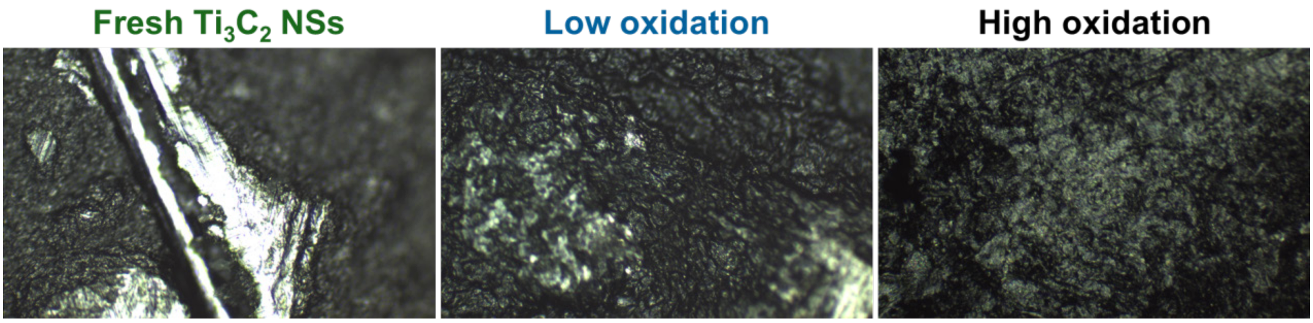

Figure S15. Raman mapping of Ti_3C_2 NSs with different degree of oxidation.

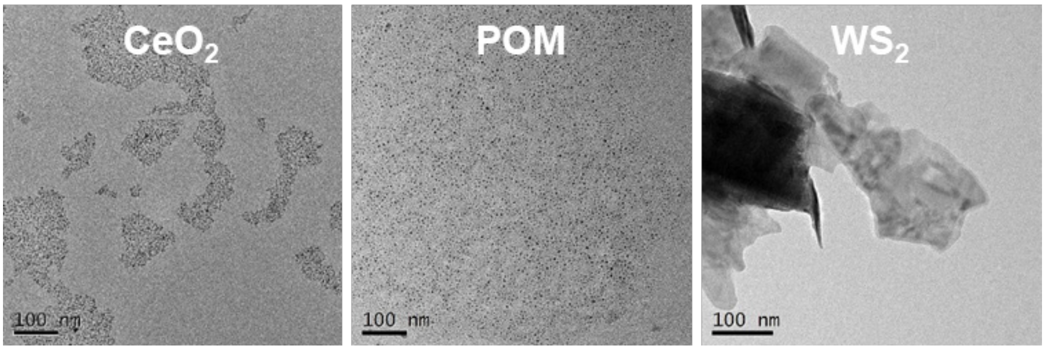

Figure S16. TEM imaging of other anti-oxidative materials containing CeO_2, POM, and WS_2.

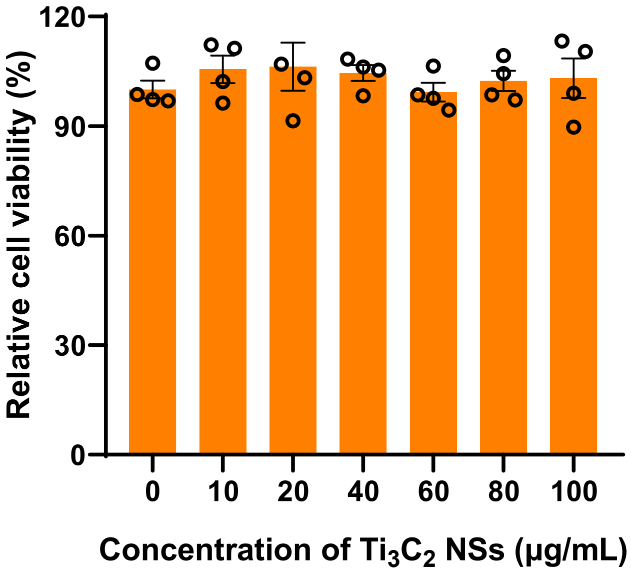

Figure S17. Relative cell viabilities of RAW 264.7 macrophage cells after incubation with different concentration of Ti_3C_2 NSs (0, 10, 20, 40, 60, 80, and 100 μg/mL).

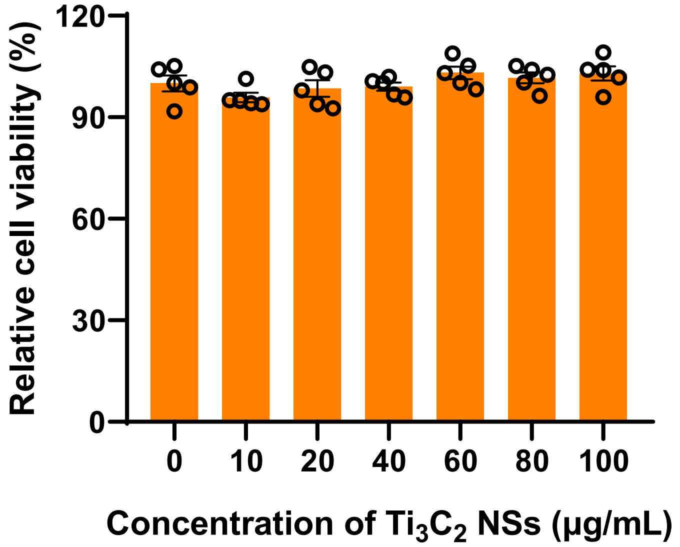

Figure S18. Relative viabilities of the HUVECs after incubation with different concentration of Ti_3C_2 NSs (0, 10, 20, 40, 60, 80, and 100 μg/mL).

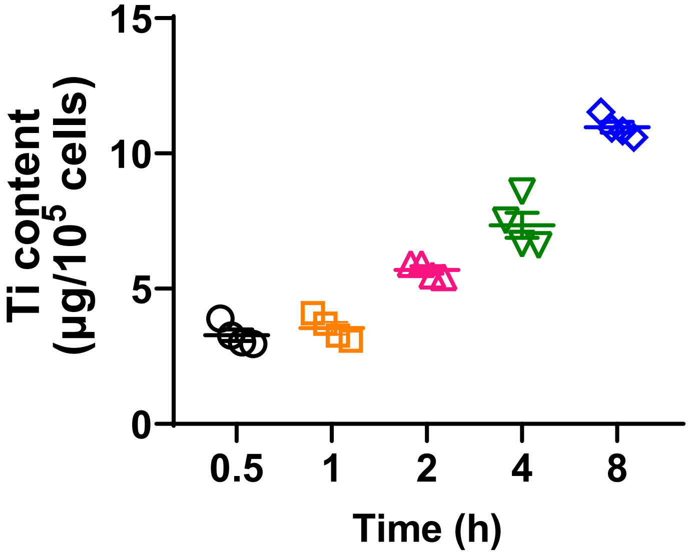

Figure S19. The HT29 cell uptake of Ti_3C_2 NSs with various incubated time measured by ICP-OES.

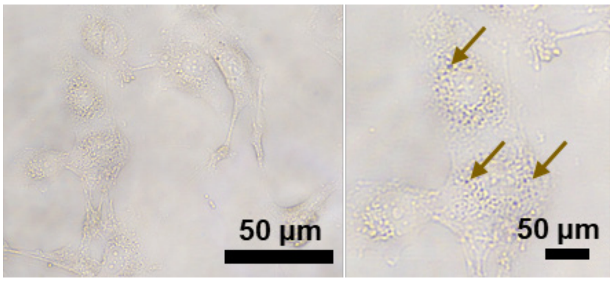

Figure S20. Microscope imaging of HUVECs after incubation with Ti_3C_2 NSs.

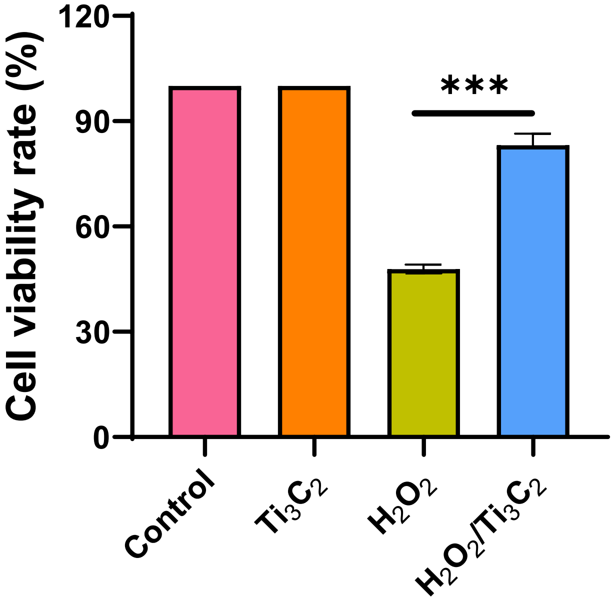

Figure S21. The ratio of live cells among all cells in the field of visions from different groups based on Figure 3E.

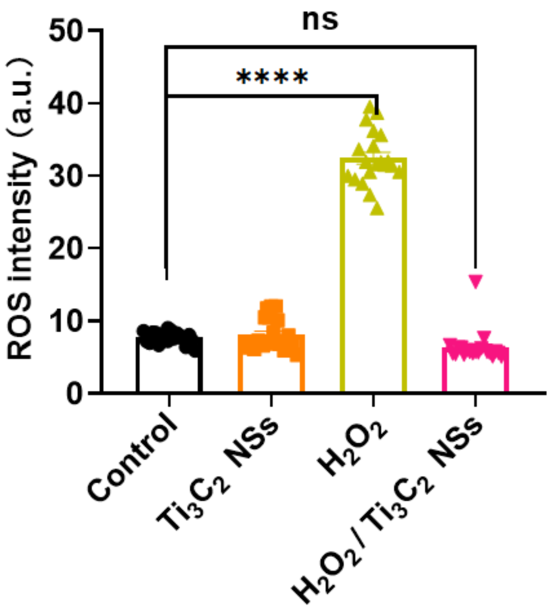

Figure S22. Statistical analysis of the intracellular ROS intensities from various groups.

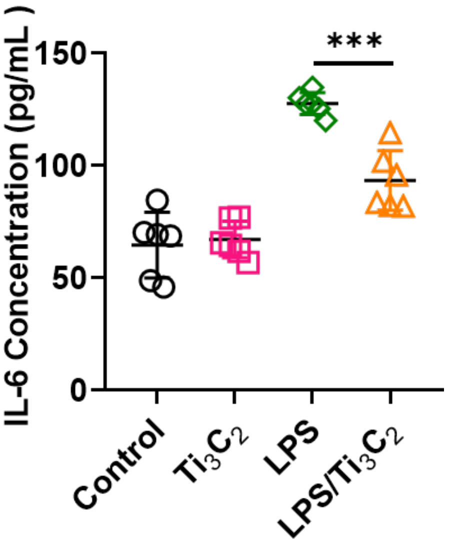

Figure S23. The expression of inflammatory cytokines from LPS-pretreated RAW 264.7 after different treatments as indicated.

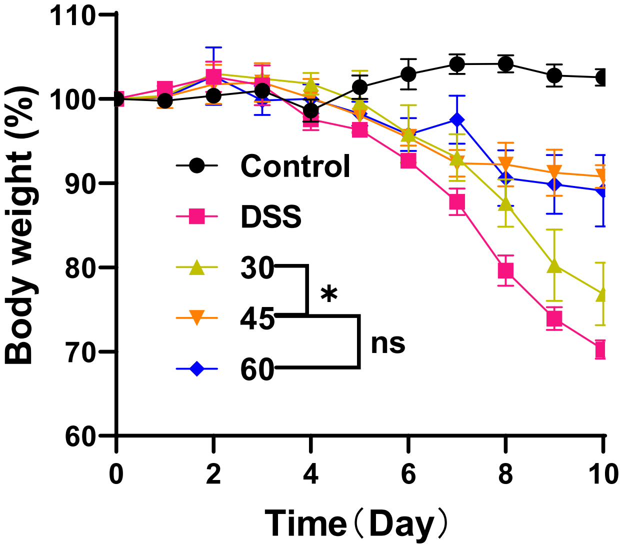

Figure S24. Comparison of the colitis therapuetic outcomes by various Ti_3C_2 NSs doses.

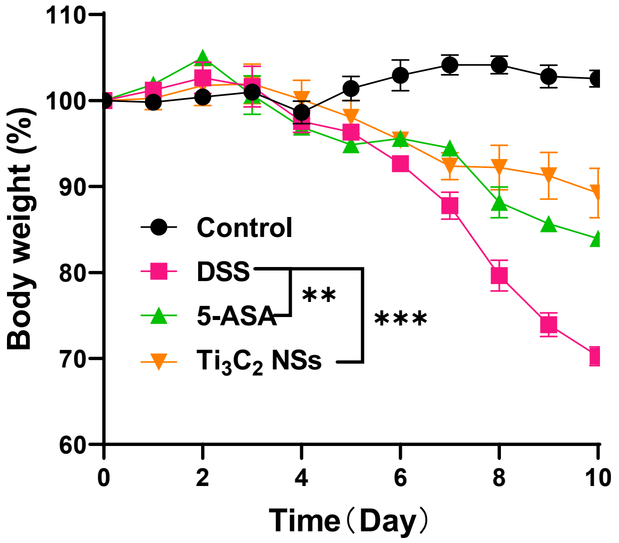

Figure S25. Daily body weight changes of mice from different groups as indicated.

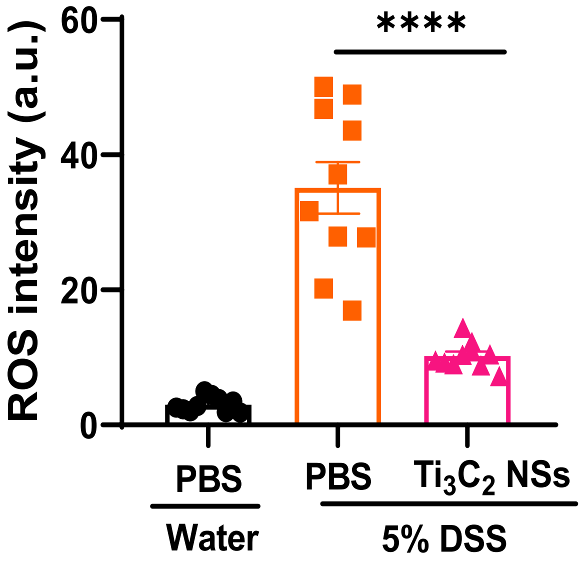

Figure S26. Statistical analysis of the ROS intensity in colon tissues after different treatments.

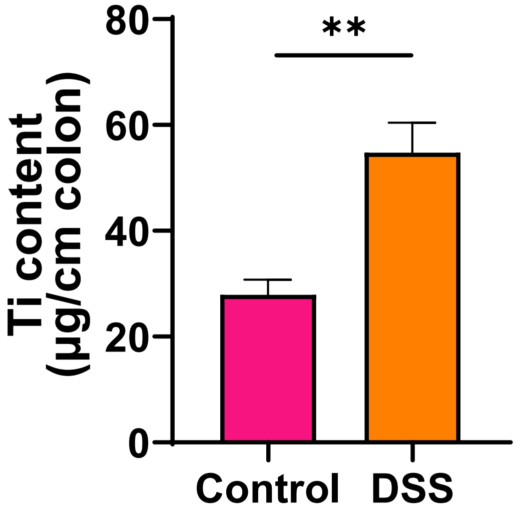

Figure S27. The comparison Ti_3C_2 NSs in the colons of normal and colitis mice.

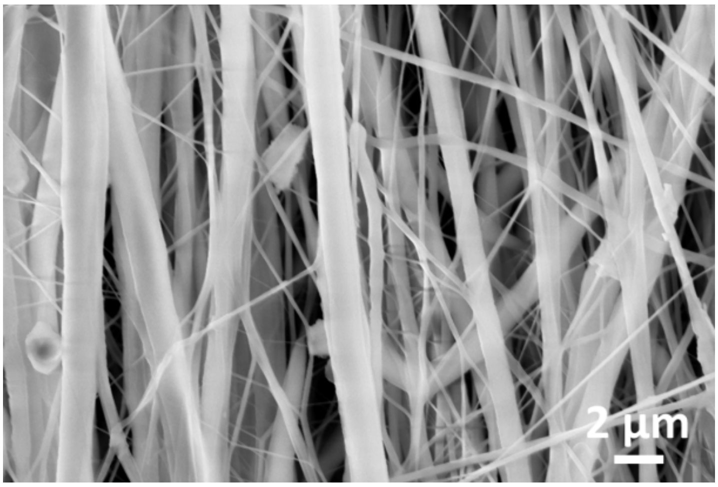

Figure S28. SEM image of PVA fibers.

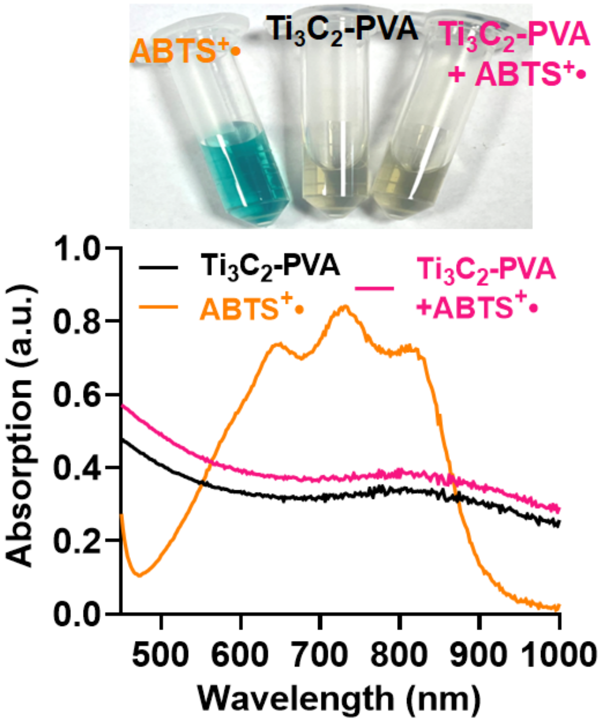

Figure S29. A photograph of ABTS+• solution after incubation with Ti_3C_2-PVA fibers.
